# Nanomaterial Solutions for Environmental Applications and Bacteriological Threats: The Role of Laser-Induced Graphene

**DOI:** 10.3390/nano15171377

**Published:** 2025-09-06

**Authors:** Mario Alejandro Vallejo Pat, Harriet Ezekiel-Hart, Camilah D. Powell

**Affiliations:** Department of Biomedical Engineering and Chemical Engineering, University of Texas at San Antonio, San Antonio, TX 78249, USA; mario.vallejo2@my.utsa.edu (M.A.V.P.); harriet.ezekiel-hart@my.utsa.edu (H.E.-H.)

**Keywords:** sensors, energy harvesting, antimicrobial, water purification, membranes

## Abstract

Laser-induced graphene (LIG) is a high-quality graphene material produced by laser scribing. It has garnered significant attention as a solution to various growing global concerns, such as biological threats, energy scarcity, and environmental contamination due to its high conductivity, tunable surface chemistry, and ease of synthesis from a variety of carbonaceous substrates. This review provides a survey of recent advances in LIG applications for energy storage, heavy metal adsorption, water purification, and antimicrobial materials. As a part of this, we discuss the most recent research efforts to develop LIG as (1) sensors to detect heavy metals at ultralow detection limits, (2) as membranes capable of salt and bacteria rejection, and (3) antimicrobial materials capable of bacterial inactivation efficiencies of up to 99.998%. Additionally, due to its wide surface area, electrochemical stability, and rapid charge conduction, we report on the current body of literature that showcases the potential of LIG within energy storage applications (e.g., batteries and supercapacitors). All in all, this critical review highlights the findings and promise of LIG as an emerging next-generation material for integrated biomedical, energy, and environmental technologies and identifies the key knowledge gaps and technological obstacles that currently hinder the full-scale implementation of LIG in each field.

## 1. Introduction

Graphene is a two-dimensional (2D) carbon allotrope consisting of a single layer of sp^2^-hybridized atoms arranged in a hexagonal honeycomb lattice. Historically, it was derived from graphite and named for its structural resemblance to materials with the “-ene” suffix, which denotes their double-bonded nature [1,2]. Renowned for its extraordinary properties, it boasts a high specific surface area (~2600 m^2^ g^−1^), electron mobility (200,000 cm^2^ V^−1^ s^−1^), thermal conductivity (3000–5000 W m^−1^ K^−1^), optical transparency (97.4%), and mechanical strength (Young’s modulus of ~1 TPa) [3,4]. Since its isolation in 2004 via mechanical exfoliation, graphene has revolutionized materials science, finding applications in electronics, optics, and sensing [5]. However, challenges in its scalable production, such as high cost and contamination risks [4,6], drive ongoing research for more efficient synthesis techniques. Fabricating graphene via laser irradiation (i.e., laser-induced graphene, LIG) is one such technique that not only circumvents the issues associated with cost and contamination but also offers unparalleled advantages compared to more conventional techniques (i.e., vapor deposition and wet chemical methods) [6,7].

### 1.1. Definition, Properties, and Applications

The laser-based synthesis of graphene is known by several terms in the scientific literature, including laser-induced nanocarbon (LINC), laser-scribed graphene (LSG), laser-written graphene (LWG), laser-derived graphene (LDG), laser-irradiated graphene (LIG), and, most commonly, laser-induced graphene or simply LIG [1]. Since its groundbreaking discovery in 2014 through the laser ablation of polyimide sheets, LIG technology has revolutionized graphene production [4,8]. LIG has emerged as a cost-effective and environmentally friendly fabrication method for producing high-quality graphene materials [6,9]. This method’s ability to create large-area graphene patterns in ambient conditions (i.e., without expensive cleanroom facilities, hazardous solvents, or complex chemical processes) eliminates the need for the complex and time-consuming fabrication procedures typically associated with traditional methods [10].

**Figure 1:**
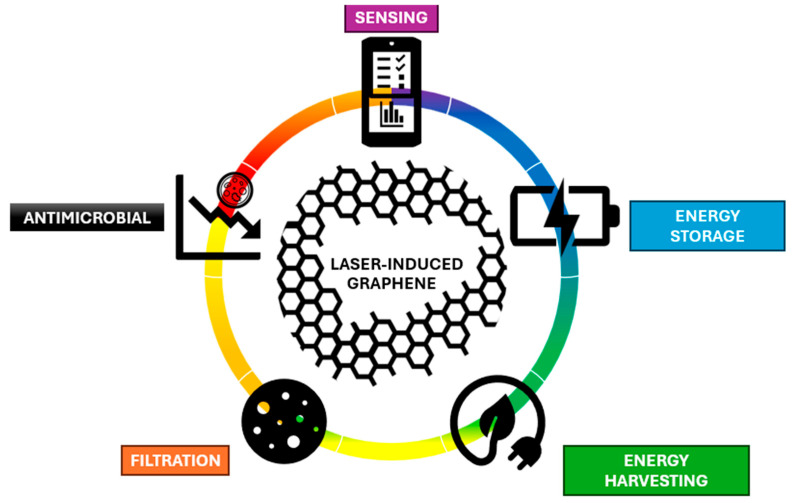
LIG for Multifunctional Applications: Sensing, Energy Storage, Energy Harvesting, Filtration, and Antimicrobial Uses.

Moreover, the simplicity of inducing graphene via laser irradiation allows for the seamless integration of graphene into electronic devices. By employing simple laser irradiation on carbonaceous precursors like polyimide, researchers can directly obtain 3D porous graphene structures in a rapid, single-step process and tunable material properties [8,11]. Moreover, by adjusting laser parameters, atmospheric conditions, additives, and substrate materials, researchers can control pore diameter, wall thickness, morphology, surface wettability, and other features to meet specific application requirements [12,13]. Again, this process can be conducted at ambient temperature and pressure using standard laser equipment, eliminating the need for high-temperature reactions, precise pressure control, or ultra-pure gases [6]. As a result, energy consumption is significantly reduced and waste production minimized, making LIG technology one of the most sustainable approaches to graphene fabrication for a variety of emerging applications (Figure 1).

Among these applications, LIG has been extensively investigated for diverse sensing schemes, demonstrating remarkable capabilities across multiple detection mechanisms, including electrochemical [9,14,15,16,17], filtration [18,19,20], and energy harvesting/storage [21,22]. These advanced sensing properties have enabled promising applications in critical areas such as physiological health monitoring, human–computer interaction systems, food safety control, and environmental pollution detection. Beyond sensing, LIG has achieved remarkable multifunctionality through various functionalization approaches, exhibiting significant potential for bactericidal [23] and antiviral [24] applications, nitrate reduction processes [25], saltwater disinfection systems [18], and other advanced technological implementations. The material’s versatility and tunable properties continue to expand its range of potential applications across multiple scientific and industrial domains.

### 1.2. Overview of Synthesis and Characterization Techniques

The morphology of LIG is controlled by three key factors: laser atmosphere, laser parameters, and substrate materials. Initially, LIG was exclusively synthesized from PI films; however, various polymers and natural materials have been identified as viable carbonaceous precursors for LIG. Over the past decade, researchers have explored numerous alternative precursors such as Polyethersulfone (PES) [26], Polysulfone (PSU), Polyphenylsulfone (PPSU) [27], Poly (PH-ddm) [28], Polyethylene Terephthalate (PET) [29], Carbon cloth–Gelatin ink [30], Aramid paper [31], Kevlar textile [32], Lignin [33], fluorinated ethylene propylene (FEP) [34], and food [35], enhancing the flexibility of synthesis parameters and broadening LIG’s applicability in various technologies. These different carbon sources influence the resulting LIG properties by introducing distinct doping components, varying ratios of pentagon–heptagon pairs, and unique planar or stereo structures [4]. 

LIG is typically synthesized under ambient atmospheric conditions, but the use of controlled gas environments (including O_2_, Ar, and H_2_) can significantly alter its surface characteristics, influencing hydrophobic and hydrophilic states [36]. The properties and performance of laser-processed carbon materials are strongly influenced by multiple laser parameters, including speed, power, height, setup configuration, optical components, and the ablation environment [12,15]. The process enables precise patterning of complex geometries through computer-aided design software, allowing for the fabrication of customized LIG structures tailored for specific applications [7]. Moreover, the doping of performance-enhancing elements into LIG mainly follows two approaches: pre-processing and post-processing doping. Pre-processing doping introduces dopants into the precursor materials before laser treatment, while post-processing modifies already formed LIG structures [37,38].

New research highlights the contribution of artificial intelligence (AI) and machine learning (ML) to the improvement of the synthesis and applications of LIG. For example, in situ Raman information combined with Bayesian optimization platforms has been used to speed up the identification of laser parameters, which results in higher-quality graphene through fewer experimental trials with lower costs [39,40]. Likewise, dual-indicator machine learning techniques enable the predictive modeling of LIG-based devices, providing interpretable insights into substrate-dependent mechanisms by linking synthesis parameters to electrochemical and structural performance [41]. Convolutional neural networks can learn adaptively to recognize and forecast the quality of LIG production within a group of substrates and laser conditions using deep transfer learning, which has also been added for real-time process monitoring [42]. This promotes scalability and reproducibility.

**Figure 2:**
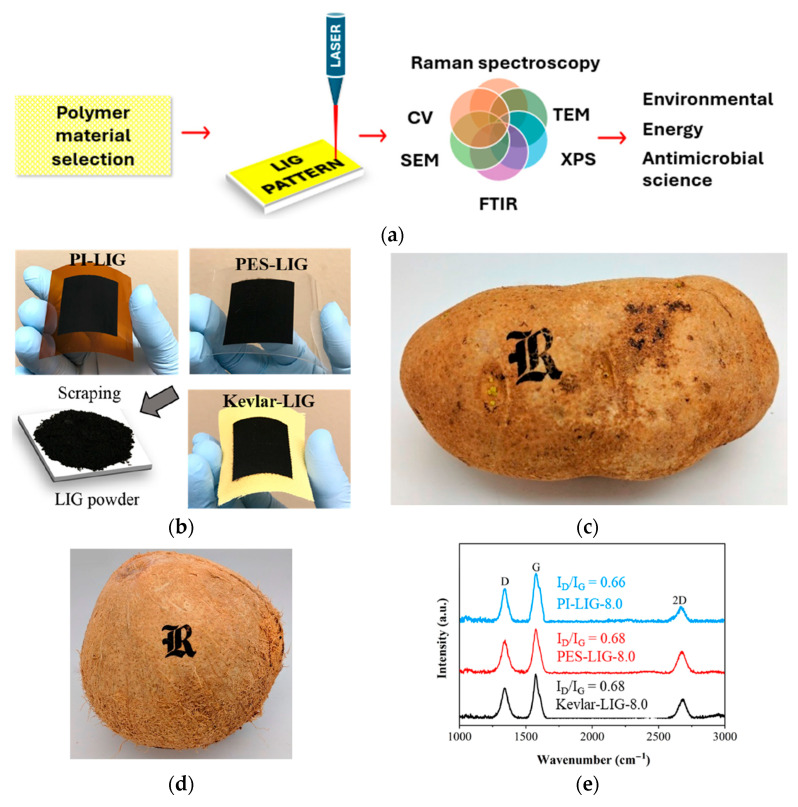

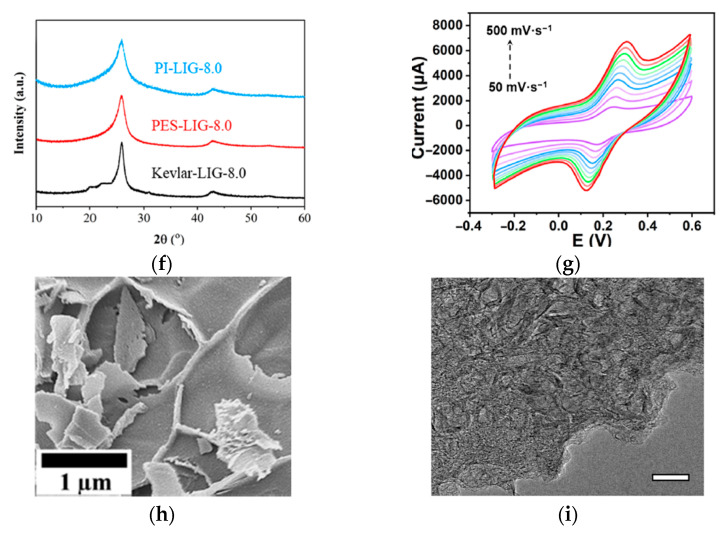
(**a**) Comprehensive schematic workflow of the LIG process, from precursor substrate selection and laser parameter optimization to material characterization and multifunctional applications. Fabrication of LIG on various polymer films: (**b**) PI, PES, and Kevlar films, reprinted (adapted) with permission from [47], copyright 2022 American Chemical Society; (**c**) potato; (**d**) coconut, reprinted (adapted) with permission from [35], copyright 2018 American Chemical Society. Characterization techniques for structural LIG: (**e**) Raman spectroscopy of few-layer graphene on PI, PES, and Kevlar substrate; (**f**) X-ray diffraction analysis LIG on Kevlar, PES, and PI, reprinted (adapted) with permission from [47], copyright 2022 American Chemical Society; (**g**) the electrochemical performance of a LIG electrode via cyclic voltammetry, reprinted (adapted) with permission from [48], copyright 2022 American Chemical Society; (**h**) SEM images of PI-LIG, reprinted (adapted) with permission from [47], copyright 2022 American Chemical Society; (**i**) low-resolution TEM image of coconut-derived LIG after five lasing passes, scale bar: 50 nm, reprinted (Adapted) with permission from [35], copyright 2018 American Chemical Society.

LIG can be characterized using a suite of techniques to evaluate its structural, chemical, electrical, and morphological properties (Figure 2). These analyses are essential for optimizing LIG’s performance in applications and obtaining a comprehensive understanding. For structural and crystallinity analysis, Raman spectroscopy is a primary tool for assessing LIG’s graphitization quality, defect density, and graphene-like characteristics. Key peaks, including the D-band (~1350 cm^−1^, indicating disorder), G-band (~1580 cm^−1^, representing sp^2^ carbon), and 2D-band (~2700 cm^−1^, confirming few-layer graphene), provide insights into the material’s crystallinity [8,9,14]. The intensity peak ratio of the D and G bands (I_D_/I_G_) helps quantify defect concentrations. X-ray diffraction (XRD) complements Raman by revealing interlayer spacing and crystallinity through peaks. For nanoscale structural details, transmission electron microscopy (TEM) can directly visualize graphene layers and defect distribution [17]. X-ray photoelectron spectroscopy (XPS) is widely used to determine LIG’s elemental composition and bonding states [43]. It also detects oxygen and nitrogen functional groups, which influence wettability and electrochemical activity. Fourier-transform infrared spectroscopy (FTIR) further identifies surface functional groups, such as hydroxyl (–OH) and carbonyl (C=O), while energy-dispersive X-ray spectroscopy (EDS) maps elemental distribution, crucial for assessing doping effects in modified LIG [44]. Scanning electron microscopy (SEM) reveals LIG’s porous, three-dimensional network, including pore size distribution and flake morphology [45]. The electrical performance of LIG is quantified using a four-point probe to measure sheet resistance and conductivity. Moreover, electrochemical performance techniques such as cyclic voltammetry (CV) evaluate charge transfer resistance, redox potentials, reaction reversibility, and reaction kinetics, which are critical for sensing and energy storage applications [46].

### 1.3. Purpose of Review

The purpose of this critical review is to systematically examine the current state of research, including synthesis techniques such as laser parameters and precursor materials, as well as structural and functional properties, and emerging applications. By assessing recent progress and ongoing challenges, particularly its advantages like scalability and versatility alongside limitations such as reproducibility and environmental stability, this review identifies key knowledge gaps and technological obstacles. Moreover, it highlights innovative developments and suggests future research directions to enhance LIG functionality, optimize performance, and facilitate its seamless integration into practical technologies. Ultimately, this comprehensive evaluation aims to steer the scientific community toward addressing current limitations and advancing the real-world adoption of LIG in next-generation devices.

To ensure a comprehensive critical review, a systematic literature search was conducted to identify relevant publications on LIG. The electronic databases Scopus, IEEE Xplore, and Google Scholar were searched from 2014 onward, with select earlier references included to provide necessary foundational context. The search strategy employed a combination of keywords and Boolean operators. The study selection process adhered to PRISMA guidelines and involved a two-stage screening procedure. First, titles and abstracts were screened against predefined inclusion and exclusion criteria (i.e., study design). Subsequently, the full texts of potentially relevant articles were rigorously assessed for final eligibility (i.e., authority and accuracy).

## 2. LIG for Environmental Applications

### 2.1. Water Purification

#### 2.1.1. Electrochemical Detection of Heavy Metal (HM) Ions

Heavy metal (HM) ions such as mercury, lead, arsenic, chromium, and copper are a hazard and threat to ecosystems due to their high toxicity, recalcitrance, and stability within the environment [9,48]. For instance, during the life cycle of resources such as water, studies have demonstrated that HMs are able to be absorbed by plant roots and subsequently by livestock as they consume said plants to ultimately cause damage to critical systems within the human body [9,37,46,49]. Fossil fuels, batteries, agricultural chemicals, paints, and mining industry activities are only a few examples of HM generators [37,49]. The FDA, CDC, EPA, WHO, and WFD have set guidelines for the maximum permissible levels in water [48,50].

**Figure 3:**
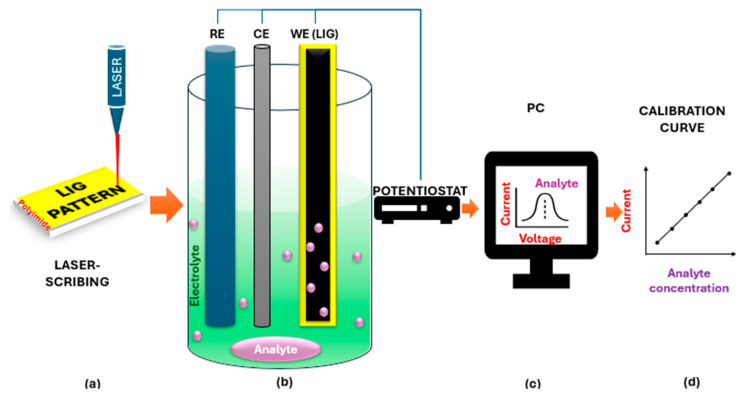
Schematic illustration of the fabrication and electrochemical performance analysis of an LIG sensor: (**a**) laser scribing of the LIG working electrode; (**b**) electrochemical characterization using a three-electrode system and potentiostat to measure analyte response in the electrolyte; (**c**) software-based analysis of potentiostatic data; (**d**) quantitative correlation of analyte concentration with current.

The detection of HMs within the environment (e.g., soil and water) is a critical measure to protect human health and the environment. Traditionally, spectroscopy-based instruments such as Inductively Coupled Plasma Mass Spectrometry/Atomic Emission Spectrometry (IC-AES), Inductively Coupled Plasma Mass Spectrometry (ICP-MS), Hydride Generation Atomic Absorption Spectroscopy (HGAAS), Graphite Furnace Atomic Absorption (GFAA), Atomic Fluorescence Spectroscopy (AFS), Atomic Absorption Spectroscopy (AAS), Ultraviolet Spectrophotometry (UVS), and X-ray Fluorescence Spectrometry (SFS) have been used to quantify HM concentrations from environmental samples [37,46,49]. Despite their high selectivity, sensitivity, and accuracy, these methods require expensive equipment, complex pretreatments and operations procedures, long detection times, specialized users, and off-site quantification [9,46,51]. In light of these drawbacks, electrochemistry-based detection methods thrive at addressing these spectroscopic challenges. Electrochemical instruments offer various advantages, such as real-time monitoring, in situ detection and quantification, cost-effectiveness, high sensitivity, easy operation, and rapid monitoring [9,46,48,51]. Suitable electrochemical techniques for HM detection include Anodic Stripping Voltammetry (ASV), Square Wave Voltammetry (SWV), Square Wave Anodic Stripping Voltammetry (SWASV), and Differential Pulse Voltammetry (DPV). These methods attain a fast and accurate detection speed with shorter potential periods compared to the conventional CV technique [9,37]. An overview of the fabrication process and performance assessment of the LIG-based sensor is illustrated in Figure 3.

Flexible materials (e.g., PI, PET, PES, and PSU) have emerged as a promising option for environmental monitoring. Conventional electrodes like carbon paste and Glassy Carbon Electrodes (GCEs) are often unsuitable for small-volume analyses due to their physical limitations, including rigidity and large sizes. Leveraging the advantageous properties of LIG, including its compatibility with other materials, LIG has become an ideal choice for flexible planar sensor devices [37,52]. Their low toxicity, limited reactivity, and strong catalytic activity make them ideal for such applications [9,46,50,53]. Additionally, metal oxides (e.g., SnO_2_ and CeO_2_) and nanoparticles (NPs) such as bismuth, platinum, silver, and gold are commonly used to modify the LIG electrode surface to improve their sensitivity, surface area, selectivity, and conductivity. However, a common interference in HM sensors arises from ion intermetallics, particularly due to the formation of Cu/Cd and Cu/Pb during deposition on the working electrode (WE) [54]. While NPs and metal oxides enhance the performance of LIG-based sensors, they present certain limitations. For instance, although Bi NPs enhance sensor performance, they are sensitive to solution pH and narrow the potential window. To address this, Jeong et al. used a pH 4.5 acetate buffer for simultaneous detection of multiple HMs [9,55]. Moreover, despite its excellent electrocatalytic activity, Pt is costly and can introduce measurement inaccuracies for HMs. Its strong catalytic properties may even reduce hydrogen ions to hydrogen gas in small amounts of water or acid [56]. Similarly, Au is unsuitable for simultaneous Pb and Cd detection due to overlapping stripping peaks, as demonstrated by Jeong et al. in their multi-HM sensing study [9,57]. Moreover, the atypical electrochemical response of As^3+^, demonstrated by Zhao et al. through SWASV measurements at 20 μg L^−1^, requires specialized detection methodologies distinct from those employed for common metal ions [37]. Despite significant advancements in sensor technology, researchers have yet to develop a universally optimized LIG-based or carbon-based electrode that can simultaneously detect multiple heavy metals with both high accuracy and freedom from the aforementioned interferences.

The properties of LIG are highly influenced by the precise optimization of laser scribing parameters during fabrication [48]. Subsequently, to ensure accurate electrochemical performance, a thorough optimization of key sensor parameters must be conducted and typically involves pH value, deposition time, deposition potential, temperature, and scan rates [9,48]. A linear calibration curve, with a proper linear correlation coefficient R^2^ and sensitivity (μA μg^−1^ L) and interference species analysis, is then acquired for each ion of interest.

Another critical parameter that significantly impacts the performance of LIG sensors for HM detection is solution pH. Numerous studies, including work by Y. Liu et al., have demonstrated that Cd^2+^ oxidation peak intensity exhibits strong pH dependence, with optimal response occurring between pH 3.6 and 5.2. Outside of this optimal range, sensor performance is compromised: at lower pH (<3.6), hydrogen evolution interferes with measurements, while at higher pH (>5.2), sulfonic amino and sulfonic acid groups preferentially undergo neutralization reactions, compromising electrode functionality [46]. This pH sensitivity presents a significant challenge for environmental monitoring applications, as natural water systems (including groundwater and surface water) often exhibit substantial variations in pH (e.g., pH ranges from 4.16 to 9.95) [58]. This pH variability requires either sample pretreatment or extensive calibration, adding complexity that reduces field applicability.

The performance of different LIG-based HM sensors is summarized in Table 1. These sensors demonstrate significant advantages, particularly in detecting single or multiple heavy metals (HMs) at low concentrations. For instance, X. Liu et al. achieved an exceptionally low detection range, with a Limit of Detection (LOD) of 0.5 μg L^−1^ for Pb ions [49]. Similarly, Ali and Zhao, and their collaborators, successfully detected individual mercury and arsenic ions by incorporating copper and gold into laser-induced graphene [37,50]. Additionally, the teams led by S.-E. Jeong and S. Jeong achieved highly sensitive simultaneous detection of Pb, Cd, and Cu ions, with sensitivities of 0.42 μA μg^−1^ L for Cu, 0.24 μA μg^−1^ L for Cd, and 0.06 μA μg^−1^ L for Pb in one study, and 0.20 μA μg^−1^ L for Pb and 0.19 μA μg^−1^ L for Cd in another analysis [9,14].

Moreover, several studies have validated LIG sensors in real environmental water samples. For instance, Y. Liu et al. demonstrated the effectiveness of LIG electrodes for Cd detection in tap and groundwater, achieving a Relative Standard Deviation (RSD) below 6.06% [46]. Similarly, Zhao et al. reported an average recovery rate of 99.2% in their analyses [37]. Further studies by S. Jeong et al. evaluated the performance of SWASV for simultaneous detection of Cd, Pb, and Cu in drinking water, showing sensitivity ranges of 0.06–0.07 μA μg^−1^ L. In tap water, the sensitivity ranged from 0.03 to 0.042 μA μg^−1^ L, highlighting the method’s robustness across different water matrices [9].

Table 1 also demonstrates the interference tolerance of various sensor platforms, with most studies reporting excellent selectivity, less than 9% of RSD. However, these assessments were conducted using simplified interference models rather than complex environmental matrices. Critical classes of potential interferents, including organic pollutants (pesticides, polycyclic aromatic carbons, phenolic compounds) and inorganic species (metal species, anions, and salts) remain uncharacterized [59]. Furthermore, Ateia and colleagues identify key commercialization challenges for sensors, emphasizing that successful field deployment requires exceptional durability and robustness in real-world operating conditions. They emphasize that sensors must be validated in real-world conditions, not merely laboratory settings, as they must maintain reliability amid variable environmental stresses, prolonged operation, and unanticipated interference factors [60]. However, to achieve this, it is essential to first identify the main substances that interfere with measurements in different types of water matrices. Hence, strategies must be developed to identify and reduce these interferences and prevent fouling resulting from the buildup of microorganisms (algae and bacteria), organic matter, and mineral scaling [61] through pore blocking and internal or cake layer formation [62] on sensor surfaces to ensure accurate and lasting operation.

#### 2.1.2. Electrochemical Immobilization of Heavy Metal (HM) Ions

Extensive research confirms that industrial wastewater frequently contains high concentrations of HM ions, which persist in the environment and endanger human health when released into water systems or soil [63]. Among these contaminants, uranium poses particular concern due to its radioactive and soluble nature. Primarily existing as hexavalent uranyl (UO_2_^2+^) in wastewater from nuclear activities (mining, fuel processing, and accidental leaks), uranium’s high solubility makes it exceptionally challenging to remove, amplifying its environmental and health risks [64,65,66]. In parallel, rare earth (RE) elements, critical for advanced technologies like magnets, phosphors, and fiber optics, also accumulate in industrial effluents [67]. Therefore, wastewater has transitioned from being classified as waste to a viable secondary resource [68].

Scientists have developed multiple approaches to recover critical elements and remove hazardous metal ion contaminants from waste batteries, industrial wastewater, radioactive water, including biological remediation, chemical precipitation, and adsorption [16,17,43,69]. However, some of these separation techniques often lead to environmental concerns, including pollution from volatile organic solvents, overconsumption of acids and bases, and chemical losses from extractants, absorbents, or eluents [70]. Electrochemical technologies have gained prominence in wastewater treatment due to their efficiency, flexibility, and sustainability advantages, requiring minimal chemicals, generating little sludge, and enabling resource/energy recovery.

Common electrochemical approaches include electrochemical oxidation (EO) for breaking down persistent organic pollutants, Electrochemical Reduction (ER) for metal recovery and nitrate removal, electrocoagulation (EC) for eliminating oils and colored compounds, and Electrodialysis (ED) or Capacitive Deionization (CDI) for desalination and water reuse [68]. In that sense, CDI or electrosorption has been widely used for recovering metal ions from wastewater due to its low cost, ease of control, and lack of secondary chemical pollution [68,71,72]. It works under an applied voltage as charged metal ions migrate toward oppositely charged electrodes via electric field forces and adsorb onto them. This process is often accompanied by simultaneous electroreduction, electro-oxidation, or electrocoagulation, depending on the specific metal ions’ redox properties [73,74,75].

More importantly, among the various technologies applied in CDI, electrode material plays a critical role. CDI electrode materials must exhibit high electrical conductivity, optimal pore size distribution, elevated specific capacitance, large specific surface area, excellent hydrophilicity, and a well-structured porous network [76,77]. LIG has gained prominence in CDI electrodes due to two key advantages: (1) its 3D porous structure for optimal pore-size-dependent adsorption, and (2) its superior electrical conductivity compared to conventionally synthesized graphene, enhancing electrochemical performance. These properties make LIG ideal for CDI and broader electrochemical applications [17,43]. Additional analytical techniques to characterize the electrochemical performance of LIG for this application include pH measurements and electrochemical impedance spectroscopy [16,43,74].

Recent studies have begun to investigate LIG technology for immobilization of heavy metals, radioactive pollutants, and recoverable rare earth elements (Table 2). In terms of traditional heavy metals, L. Wang and colleagues demonstrated the recovery of Co^2+^, Cd^2+^, and Ni^2+^ from nitrate and sulfate solutions using LIG electrodes via electrosorption and electrodeposition. Upon voltage application, solution pH decreased while conductivity increased, with Cd^2+^ exhibiting superior reactivity (Cd^2+^ > Co^2+^ > Ni^2+^) due to its lower standard potential and stronger reducibility to ultimately yield a higher adsorption capacity (Cd^2+^ > Co^2+^ ≈ Ni^2+^). Moreover, recovered Co(OH)_2_ was successfully repurposed into a LiCoO_2_ cathode, delivering a practical specific capacity of 122.8 mAh g^−1^, highlighting the method’s potential for sustainable metal recovery and green energy applications. In Table 2, the maximum adsorption capacities (mg g^−1^) for Cd^2+^, Co^2+,^ and Ni^2+^ were 3479.8, 1381.5, and 1448.7, respectively, as summarized [16].

In terms of radioactive pollutants, Sun and coworkers demonstrate a straightforward titration hydrolysis method to modify LIG with hydrated titanium oxide (HTO), significantly enhancing its adsorption capabilities (Table 2) in terms of capturing this radioactive pollutant commonly found as UO_2_^2+^ within the environment [65,66]. Their LIG/HTO composite shows remarkable improvements in both proton transfer and specific capacitance (4.74× higher than pure LIG). As a CDI electrode, it achieves an exceptional uranium adsorption capacity of 1780.89 mg/g at 1.2 V. The electro-adsorption mechanism involves the formation of UO_4_·4H_2_O (with O^2−^). These dual chemical and electrostatic interactions enable highly efficient uranium capture [17].

Similarly, in the study reported by Cao et al., a novel LIG/Co_4_S_3_ electrode fabricated by combining LIG with electrodeposited cobalt sulfide (Co_4_S_3_) for highly efficient uranium removal was presented. The optimized LIG called *LIG_6_/Co_4_S_3_-15* electrode exhibits dual charge storage mechanisms (electrical double layers and pseudocapacitance), achieving a specific capacitance of 24.27 F g^−1^, 1.82 times higher than bare LIG_6_ (13.36 F g^−1^) and an exceptional UO_2_^2+^ adsorption capacity of 2702.79 mg g^−1^. The electrode’s performance stems from its hierarchical structure: the porous, conductive LIG framework facilitates rapid charge/ion transport, while the layered Co_4_S_3_ nanosheets provide hydrophilicity and abundant active sites. Uranium removal occurs via a three-step mechanism: (1) initial electrosorption onto the electrode surface, (2) physicochemical adsorption onto the porous matrix, and (3) electrocatalytic reduction/deposition onto a stable solid phase [43].

In the case of REs, one study demonstrates the effective recovery of La, Nd, and Ce from aqueous solutions using LIG films as electrodes through an electrosorption technique. The process, influenced by applied current, voltage, and initial concentration, significantly enhanced desorption capacities, achieving remarkable values of 2510.5 mg g^−1^ for La, 2349.25 mg g^−1^ for Nd, and 2150.75 mg g^−1^ for Ce, surpassing conventional physical adsorption materials. The recovery mechanism, driven by both electrostatic attraction and electric force, yielded RE precipitates in the forms of La(OH)_3_, Nd(OH)_3_, and CeO_2_ [74].

Unfortunately, current research on LIG technology for HM immobilization and resource recovery remains limited. However, recent trends in wastewater treatment have shifted from pollution remediation to resource recovery, particularly for heavy metals [71]. Recovering these metals not only mitigates environmental pollution but also provides a sustainable source of valuable materials, offering both engineering and ecological benefits [71,78]. Despite these advantages, as noted by Liu and coworkers, electrochemical technologies for resource recovery from wastewater have received relatively little attention. Moreover, researchers have largely overlooked the Faradaic efficiency and selectivity of these systems under competing ions, creating a significant knowledge gap. Yet, these methods hold significant promise: based on the principles of electrochemical reaction (without chemical additives), they can effectively recover metallic ions, nutrients, sulfur, hydrogen, and other valuable compounds [68]. Given these opportunities, further research is needed to explore LIG as an electrochemical technology, leveraging its unique properties to advance sustainable wastewater treatment and resource recovery.

#### 2.1.3. Filtration Systems

**Figure 4:**
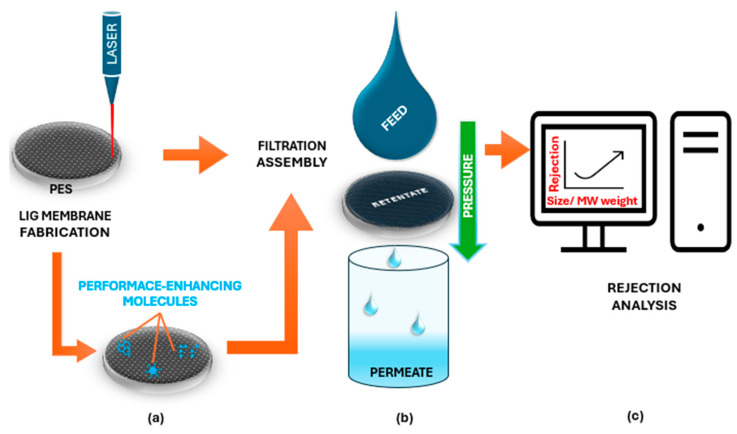
Schematic illustration of the LIG membrane process, including (**a**) fabrication via laser irradiation with optional incorporation of performance-enhancing molecules; (**b**) a cross-flow filtration assembly highlighting key components: feed, retentate, and permeate; and (**c**) the analytical methodology used to evaluate solute rejection and membrane selectivity.

The global water crisis continues to escalate, making innovative methods for producing potable water increasingly vital. As freshwater resources diminish or become contaminated, alternative sources such as seawater and wastewater must be purified via sustainable technologies to ensure safe consumption [26,79]. Membranes are widely used in aqueous physical separation processes (i.e., filtration) for applications such as HM ion removal, oil–water separation, emerging contaminant elimination, and desalination [18,19,80]. PES porous membranes are cost-effective, easily converted into graphene via lasing, and widely employed in water purification applications. These PES-based LIG porous filters can be fabricated via a single-step process, offering facile scalability [26]. This LIG technique has been successfully applied to various asymmetric microporous membranes fabricated from aromatic polysulfone-class polymers, including PSU, PPSU, and PES [27]. In particular, LIG-PES composite membranes have demonstrated excellent functional properties for filtration applications, which include enhanced electrochemical performance, improved electrical conductivity, and notable antifouling and antimicrobial characteristics (Figure 4) [23,26,27].

Unfortunately, these superior properties are only achievable through optimal fabrication process parameters [20,26]. The poor mechanical stability of graphene-based membranes remains a significant obstacle to their practical application in filtration systems [20]. Studies report the fragility of the LIG layers formed on membrane surfaces, which are prone to damage under operational conditions. Thus, developing mechanically robust LIG membranes presents an ongoing challenge, as laser irradiation often distorts the porous substrate structure, limiting their filtration performance and applicability. To address this issue and increase the device robustness, LIG is transferred onto materials including PDMS, epoxy, or polypropylene by infiltrating them into freshly formed porous LIG on PI, using gravity or hot pressing. After curing, the structure is flipped, and the PI layer is removed to expose the composite surface [81,82].

In addition to the common surface and morphological characterization tools used for LIG, LIG-based membranes have their physical and mechanical characteristics evaluated through various tests (i.e., pore size distribution, mechanical strength, and ablation resistance). Specifically, surface properties are examined via contact angle measurements, and performance is evaluated based on separation efficiency; water permeance, also called liter of permeate per square meter of membrane area per hour (LMH); antifouling behavior; and reusability. Additionally, computational insights are obtained through Visual Molecular Dynamics simulations [13,20,23,80,83].

##### Enhancing LIG-Based Membrane Filtration Performance (BSA Rejection)

Table 3 and Figure 4 present an overview of LIG-based membranes developed for BSA filtration applications. Multiple authors, including Thakur, Singh, Thamaraiselvan, and Kleinberg, have investigated various strategies to address the inherent limitations of LIG membranes. These approaches include the incorporation of functional additives such as (1) graphene oxide (GO) to enhance sensor performance [23,50]; (2) Polyvinyl Alcohol (PVA), which contributes biocompatibility, non-toxicity, and excellent film-forming properties [13,84]; (3) Glutaraldehyde (GA), as an effective crosslinking agent [13,23]; or (4) glycerol, which functions as a pore-preserving agent during membrane fabrication [20]. Along these lines, these modifications have collectively advanced the performance characteristics of LIG-based filtration systems. For example, the researchers mentioned above developed a robust hybrid LIG-GO membrane by crosslinking and filtering GO onto a LIG support to create an ultrafiltration membrane. Increasing the GO content enhanced rejection rates to 69% for BSA and 99.9% for bacteria, correlating with a measured molecular weight cutoff (MMWC) of ~90 kDa, while simultaneously improving antifouling properties with 83% less biofilm growth compared to conventional polymer membranes. Additionally, the LIG substrate retained its electrical conductivity, enabling the membrane to function as an antimicrobial electrode that eliminated bacterial viability when electrified. In this configuration, applying a 3 V anodic electric field during cross-flow filtration tests improved the system’s water flux by 11% as compared to traditional ultrafiltration membranes [23].

In similar studies, an electrically conductive LIG-PVA composite membrane for ultrafiltration was developed upon adding PVA 4% *w*/*v* to the LIG surface. Said LIG-PVA membranes were shown to block 63% more BSA and nearly all bacteria (99.9%), matching ~90 kDa MMWC as compared to LIG with PVA concentrations from 0.5 to 3% *w*/*v*. The mentioned LIG-PVA membranes also exhibited antibiofilm property through the electrical killing effect when a lower PVA content was used [84].

Another investigation reported that glycerol treatments preserved the nanoscale properties of LIG-PVA despite one-sided lasing (bottom or top), mechanical agitation (sonication), and variable chemical conditions (acidic and alkaline solutions). And, when using 8% laser power on the PES bottom layer, the resulting membrane, called *PES(B)-LIG-HP* (B = bottom, HP = high power), demonstrated superior performance, achieving 865 LMH flux (4× higher than UP010 commercial membrane) while maintaining 90.9% BSA rejection [20].

Although these functional additives enhance certain membrane properties, they introduced critical performance trade-offs: (1) higher laser power boosted water flux but compromised BSA rejection [20], (2) increased PVA concentration improved LIG pore coverage but reduced effective pore size and flux [84], and (3) elevated GO content diminished water permeance due to added hydraulic resistance [23]. These inverse relationships demonstrate that optimizing membrane performance requires careful balancing, necessitating additional research to define ideal properties for protein filtration applications.

##### LIG-Based Membranes for Thermally Enhanced Filtration Techniques

Mahbub et al. developed improved LIG membranes for desalination by combining RF heating with CaCl_2_ salt addition, where CaCl_2_ actually played a key role in maintaining membrane porosity during drying [18]. The RF heating approach offers distinct advantages for membrane distillation—its non-contact operation, deeper material penetration, and safer low-frequency operation make it superior to Joule heating (JH), induction heating, or microwave heating [18,86]. The authors found that the hydrophobic character of the membrane was verified by its surface water contact angle (143.7°). Additionally, the membrane’s thermal profile reached surface temperatures of ~140 °C at an optimized RF frequency and power (i.e., 91 MHz and amplifier gains of 10% (~15 W), respectively), confirming the membrane’s suitability for thermal membrane distillation (TMD) applications. They also evaluated the synergistic effect of combining vapor membrane distillation (VMD) with integrated RF heating for four days, finding that the membrane achieved an exceptional permeate flux of 13.5 L m^−2^ h^−1^ while maintaining >99% salt rejection.

Tan and coworkers employed an alternative approach for membrane distillation by developing an innovative Janus membrane by coating PES with PDMS in limonene (eco-friendly solvent) and laser-treating the active layer. The design enabled efficient JH via electrodes connected to the LIG surface using standard alternating current power. This configuration provided a layer that minimized thermal losses, significantly enhancing TMD performance. Compared to conventional pre-heated feed systems, the Janus membrane achieved 119.5% higher flux, 53.9% lower specific heating energy, and 119.5% greater heat utilization, all while maintaining excellent structural stability [85]. These alternative thermal-driven technologies offer sustainable advantages beyond common TMD through PDMS PES membranes.

##### LIG-Based Membranes for Filtration of Complex Contaminant Water Mixtures

Table 2 also summarizes the use of LIG membranes for treating complex mixtures or removing multiple impurities simultaneously (e.g., methylene blue dye and salt removal). One key investigation focused on fabricating LIG on porous PES membranes (commercial UP010 and lab-cast PES15) through laser parameter optimization. The resulting *UP010-ID7 LIG* filters exhibited outstanding performance capabilities: (1) desalination via Interfacial Evaporation (IE) achieved rates of ~1.1, 1.8, and 2.82 kg m^−2^ h^−1^ in single-, double-, and triple-stacked configurations under JH (with solar heating also evaluated); and (2) water purification showed ~100% dye removal and complete 6-log bacterial inactivation at 5 V (outperforming non-optimized filters at 2.5 V) [26].

Moreover, researchers have been increasingly utilizing activated carbon fibers (ACFs) in separation membranes for their high porosity and surface modifiability to achieve dual superhydrophobicity/superoleophilicity properties for selective oil–water separation. This is also the case with Gupta’s research group. They have shown a LIG/ACF membrane—synthesized by drop-casting a phenol–formaldehyde copolymer—with an exhibited superhydrophobicity (water contact angle: 155.4 ± 0.3°) and superoleophilicity (oil contact angle: ~0°) for exceptional separation performance for water-in-oil emulsions (5% *v/v*, 3 bar) with an efficiency over 99% and an initial flux of 771 ± 39 L m^−2^ h^−1^. The membrane also demonstrated a robust chemical stability within acidic, alkaline, and corrosive conditions [19].

Another study focused on iohexol and Cr (VI) as the pollutant targets for LIG engraved on PES. The system achieved 90% Cr (VI) removal within 6 h (exceeding 95% after 8 h) at 3 V and pH 2, with XPS and EDX analysis confirming reduction primarily to Cr (III) alongside minor Cr (I) and Cr (0) deposition on the electrode membrane. Simultaneously, 50% iohexol degradation occurred within 6 h, yielding seven identifiable transformation products (scan ranges 695, 570, and 443 *m/z*) through oxidative deiodination, amide hydrolysis, and deacetylation pathways, as characterized by UPLC-MS. The process demonstrated cost-effectiveness with an electrical energy consumption of ∼$0.08 m^−3^ for treating 2 mg L^−1^ Cr (VI) solutions [80].

In terms of complex wastewater mixtures, Gupta et al. developed a scalable method for fabricating conductive LIG-PVA composite membranes by coating and crosslinking PVA/glutaraldehyde on LIG. The 2.5% PVA-LIG membranes exhibited high permeability (900–1300 LMH/bar), mechanical-thermal stability, and effective wastewater treatment by effectively (1) reducing turbidity to 0.60 NTU, (2) lowering the Chemical Oxygen Demand/Biochemical Oxygen Demand by 10–25-fold, and (3) achieving enhanced bacterial removal at 2.5 V [13]. Similarly, Singh et al. demonstrated that PES-LIG membrane electrodes achieved complete bacterial inactivation (6-log reduction) within a flow-through filtration mode, operating at 500 LMH (2.5 V) and 22,000 LMH (20 V). Both studies highlight the potential of LIG-based membranes for scalable water purification schemes, with broader applications in the fields of biomedical, electronic, and environmental [27]. While novel materials offer enhanced functionalities as previously discussed, challenges like a lack of techno-economic analysis, limited long-term durability data, in situ testing and analysis, and slower manufacturing speeds hinder widespread adoption.

### 2.2. Energy Storage and Harvesting

#### 2.2.1. Energy Storage

The crucial shift from fossil fuels to renewable energy, spurred on by the technological advancements of electrification and global warming concerns, has driven a surge in demand for advanced energy storage solutions. Within this field, rechargeable batteries emerge as a leading option due to their high energy density, specific surface area, efficiency, reversibility, portability, and low maintenance costs (particularly so in key sectors like smart grids and electric transportation) [87,88,89,90,91]. Current research has significantly advanced the development of LIG for battery and supercapacitor (SC) applications over the past ten years.

**Figure 5:**
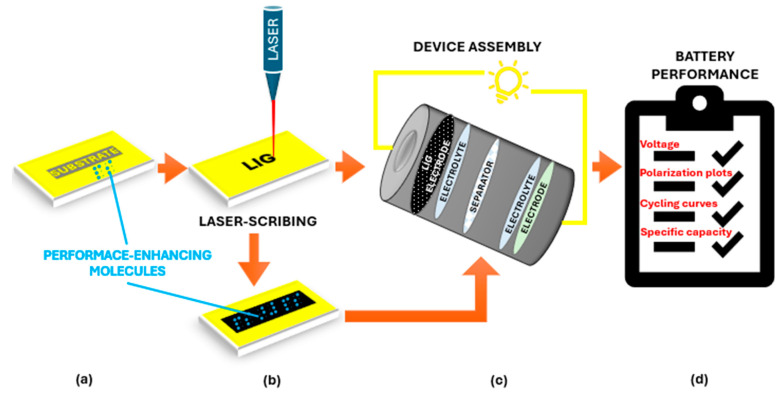
Comprehensive graphical representation of the fabrication and electrochemical evaluation protocol for LIG-based energy storage devices: (**a**) precursor substrate selection, including optional integration of performance-enhancing molecules; (**b**) laser scribing process parameters and post-synthesis functionalization strategies, such as the addition of performance-enhancing dopants; (**c**) schematic of device assembly showing the configuration of LIG electrodes, electrolytes, separators, and current collectors in a two-electrode configuration; and (**d**) electrochemical performance metrics, including voltage profiles, polarization curves, galvanostatic charge–discharge cycling stability, and specific capacity measurements.

The initial characterization of LIG for battery and supercapacitor applications involves a suite of analytical techniques to assess its structural, chemical, and electrochemical properties. XPS probes surface chemistry and bonding states, the Brunauer–Emmett–Teller (BET) analysis quantifies porosity and specific surface area, whereas TGA evaluates thermal stability. Taken together, these techniques, along with those previously mentioned, provide a comprehensive understanding of LIG’s suitability for advanced energy storage systems [11,92,93]. A schematic depicting the fabrication and performance evaluation of LIG energy storage devices is shown in Figure 5.

##### Batteries

LIG materials show great promise for enhanced battery performance due to their scalable, energy-efficient, and cost-effective production compared to traditional graphene synthesis methods like mechanical exfoliation or chemical vapor deposition. This technique enables maskless, ambient condition fabrication of binder-free and additive-free electrodes. LIG offers advantages such as a 3D porous structure, heteroatom doping, tunable mechanical properties, flexibility, high conductivity, and compatibility with various substrates [87]. When employed in lithium-ion batteries (LIBs) and sodium-ion batteries (SIBs), these advantages collectively improve the insertion and diffusion of lithium and sodium ions, particularly during ultrafast charging and discharging, while simultaneously achieving a high energy storage capacity and rapid, stable charge response [94,95]. Moreover, the high number of nucleation sites and low local current density promote the formation of smooth, dendrite-free lithium deposits, thereby improving the stability of lithium metal anodes in batteries (LMBs) [22]. LIG and its composite or modified materials are primarily used in LIBs and SIBs as electrodes, as well as in LMBs as current collectors. Their corresponding applications are summarized in Table 4.

Studies demonstrate the promising applications of LIG in advanced battery technologies. Aslam et al. [92] developed a self-supporting LIG foam anode for lithium-ion batteries, achieving an initial areal capacity of 203 µAh cm^−2^ that increased to 280 µAh cm^−2^ after annealing, with stable cycling performance (i.e., ~99% Coulombic efficiency, also called CE for over 100 cycles) and improved conductivity. Similarly, Yi et al. created a 3D hierarchical LIG composite on polyimide/copper substrates that stabilized lithium metal anodes, enabling overpotential-free Li nucleation and a high CE of around 99%, with full cells maintaining 90% capacity after 250 cycles [96]. Additionally, advancements in LIG composites have significantly enhanced battery performance through strategic material integration. Zhou et al. [97], for example, developed MnO/Mn_3_O_4_/N-doped graphene hybrid anodes via one-step laser scribing, achieving Li-ion storage (992 mAh g^−1^ at 0.200 A g^−1^) and a CE of 67.3% after 35 cycles. This relatively low initial efficiency reflects the challenges associated with incorporating metal oxides into graphene matrices and highlights the need for further optimization of the MnO/Mn_3_O_4_ nanoparticle distribution. The results suggest that while the composite architecture shows potential for high-capacity applications, careful nanostructural engineering is required to improve charge transfer efficiency and reduce irreversible capacity losses in early cycles.

For example, Cho et al. improved interface properties on Al foils, which enhanced rate performance and thermal stability in Lithium Nickel Manganese Oxide/Lithium Titanium Oxide with full cells, revealing a discharge capacity of 115.7 mAh g^−1^ at 1 C [98]. X. Xu and coworkers [99] created covalent-bridged SnS_2_/graphene heterostructures with a sodium-ion storage capacity of 597 mAh g^−1^ at 0.200 A g^−1^ after 200 cycles through laser-manufactured metastable NPs. For lithium metal batteries, Xiao et al. designed lithiophilic MnOx-decorated LIG that enabled dendrite-free operation at ultrahigh current density (40 mA cm^−2^) and capacity (20.0 mAh cm^−2^) [100]. Fang et al. further demonstrated a 3D graphene-supported Ge anode via laser ablation, overcoming volume expansion issues while achieving a capacity (860 mAh g^−1^ over 200 cycles) in both half- and full-cell configurations [93]. These studies collectively highlight LIG’s versatility as a platform for advanced battery materials when combined with oxides (MnOx), metals (Ge), and chalcogenides (SnS_2_), offering solutions for both Li-ion and next-generation battery systems through tailored nanostructuring and interfacial engineering.

##### Supercapacitors (SCs)

To meet the energy demands of portable and wearable devices, as well as flexible microelectronics development of advanced micro-energy storage systems is essential, with SCs emerging as a leading solution due to their high specific capacitance, exceptional power density, fast charge/discharge rates, and long cycle life. SCs are classified into two main types based on their energy storage mechanisms: carbon-based capacitors, which rely on physical electrostatic charge storage at the electrode/electrolyte interface, and pseudocapacitors/Faraday quasi-capacitors (PCs/FQCs), which utilize reversible redox reactions in transition metal oxides, hydroxides, or conductive polymers. Among electrode materials, LIG offers distinct advantages, including a high specific surface area that enhances active carbon sites, expands the Helmholtz double-layer region for improved ion adsorption, and ultimately boosts specific capacitance, making it a highly promising material for next-generation flexible supercapacitors [11,101]. As summarized in Table 5, the two-electrode geometry of SCs employs LIG patterned in one of two primary architectures: planar (sandwich) [33] or interdigitated [102].

Recent advances in LIG technology have led to the development of enhanced LIG-derived materials through strategies such as heteroatom doping, integration of pseudocapacitive materials, and utilization of novel carbon precursors, significantly improving the performance of flexible SCs, as detailed in Table 5. Liu and colleagues [107] developed ultrahigh-energy, flexible in-plane hybrid micro-supercapacitors (IHMSCs) using Fe_3_O_4_ nanoparticle-anchored porous LIG. As shown in Table 5, the device demonstrated the highest capacitance at 644 mF cm^−2^ at 1.0 mA cm^−2^, as compared to the other devices, and good mechanical flexibility. However, it performed with a poor capacity retention and cycling number (i.e., 74.0% and 900, respectively).

This LIG/Fe_3_O_4_ composite was fabricated in a single laser deposition step, integrating Fe_3_O_4_ NPs onto a macroporous LIG framework. Characterization analyses confirmed well-dispersed Fe_3_O_4_ NPs with mesopores on the LIG scaffold. The composite exhibited superior wettability and capillary action, enhancing ion transfer kinetics. Increasing Fe_3_O_4_ loading boosted capacitance, with the optimized device *(LIG/Fe_3_O_4_//LIG-300-IHMSC)* achieving an energy density of 60.20 μWh cm^−2^ at 1.0 mA cm^−2^. These results surpass the values reported by Liu et al. [117]. On the other hand, Basu and colleagues developed a flexible interdigitated microsupercapacitor using CO_2_ laser direct writing to convert Co-MOF (ZIF-67) on PI into what they named laser-induced MOF-derived graphene. This long-wavelength photothermal process yielded an interconnected graphitic mesoporous carbon network with several properties, including catalytic cobalt incorporation, nitrogen doping, and oxygen-rich surface groups from ambient processing. As a result, the fabricated device demonstrated exceptional stability, retaining ~99% capacitance over 200,000 cycles [38].

Demonstrating the versatility of LIG-based energy storage systems across different substrate materials, multiple research groups (Cao et al., Zhao et al., Lin et al., Lu et al., Wang et al., Correia et al., and Rao et al.) have successfully synthesized LIG from various polymeric substrates. Comparative analysis of these studies reveals consistent electrochemical performance, with reported capacitance retention exceeding 85.1% after 5000 charge–discharge cycles. The fabricated devices exhibited areal capacitances spanning a broad range from 1.66 to 125.35 mF cm^−2^ [28,29,30,31,32,113,114]. Employing an alternative approach to obtain a nitrogen (N) and sulfur (S) doped LIG SC, Du et al. fabricated a high-performance supercapacitor by laser-scribing graphene from a lignin/sodium sulfate precursor onto a flexible PET film. They assembled an asymmetric supercapacitor using the LIG as the cathode, a poly(3,4-ethylenedioxythiophene) (PEDOT)-modified LIG anode, and a flexible polymer electrolyte separator. This device achieved a specific capacitance of 29.9 mF cm^−2^ [33].

By incorporating an alternative substrate, poly(furfuryl alcohol) or PFA, Hawes et al., developed a device which demonstrated remarkable stability. Their device maintained 103% of its initial capacitance after 12,000 cycles, with their capacitance value rising from 9.50 mF cm^−2^ to 9.79 mF cm^−2^. The authors attribute their >100% capacitance retention to several factors. First, the salt surface coating possibly dissolved into the gel electrolyte to marginally boost capacitance. Second, since the device was unsealed and exposed to ambient laboratory conditions, fluctuations in temperature and humidity potentially impacted its performance. And third, a gradual infiltration of the electrolyte into the LIG pores might have also played a role in the observed capacitance increase over time. A similar rise in capacitance was noted in devices that were left idle without cycling, indicating that changes were likely due to the electrolyte rather than device degradation. Moreover, the nearly identical discharge curves recorded at cycles 1000, 2000, 10,000, and 12,000 underscore the stable cycling performance of the LIG-based supercapacitors utilizing PFA with 25% (*w/w*) Na_2_SO_4_ [115]. On the other hand, Ai et al. observed similar behavior, a capacitance of 130% of the initial value. Here, the authors stated that the behavior was potentially attributed to the incomplete saturation or contact between the PVA/H_2_SO_4_ hydrogel as the electrolyte and electrode [117].

Although existing studies provide valuable insights into LIG for batteries and supercapacitors, most foundational research on LIG integration dates back nearly a decade. Recent work has primarily focused on enhancing LIG properties through elemental doping, but these improvements are typically evaluated under idealized conditions (e.g., controlled temperature). In real-world applications, however, devices operate in non-ideal environments where factors like temperature critically impact performance. For batteries, elevated temperatures accelerate solid electrolyte interphase growth, thermal runaway, and electrolyte decomposition [120,121], while low temperatures increase internal resistance and impede Li^+^ diffusion [122]. Similarly, supercapacitors suffer from organic electrolyte degradation at high temperatures [123] and reduced ionic conductivity at low temperatures [124]. Additional research gaps become apparent when examining charge/discharge cycles have been reported to battery systems risk of cathode oxidation from overcharging [125].

Moreover, analysis of safety considerations involves cycling stability issues, where repeated charging can lead to hazardous degradation mechanisms. For example, particle cracking during cycling may progress to thermal runaway and potential explosion risks [94]. This disconnection between idealized laboratory studies and practical operating conditions represents a significant barrier to commercialization. Future research must prioritize performance evaluation under realistic environmental and operational stresses to facilitate the transition from promising laboratory results to viable commercial applications.

#### 2.2.2. Energy Harvesting

**Figure 6:**
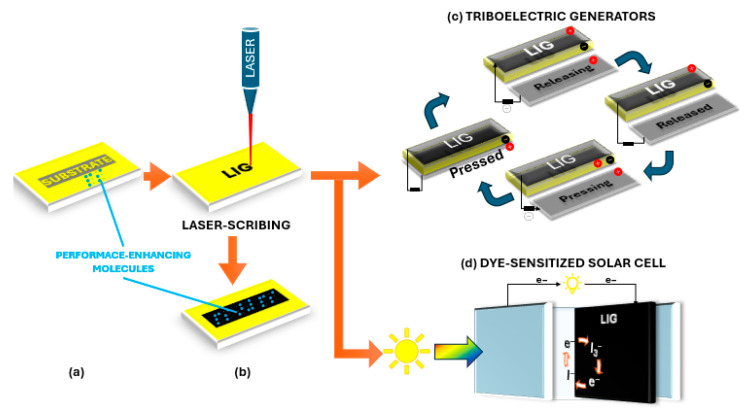
Integrated workflow for the design, fabrication, and operational mechanism of multifunctional LIG-powered energy harvesting systems: (**a**) precursor substrate selection and optional integration of performance-enhancing molecules; (**b**) laser scribing process with optional dopant functionalization to tailor electrical and triboelectric properties; (**c**) triboelectric nanogenerator operation cycle illustrating continuous energy generation during pressing and releasing; and (**d**) dye-sensitized solar cell architecture and electron transport mechanism, featuring photoexcitation, electron injection into the semiconductor layer, external circuit current flow, and electrolyte-mediated dye regeneration via an iodide/triiodide redox couple for sustained power output.

The progress of flexible electronics is intrinsically linked to advances in sustainable power solutions, making energy harvesting technology increasingly vital for powering adaptable devices and wireless systems [22]. As the world increasingly prioritizes renewable energy sources to meet global demands sustainably, there is a critical need for power solutions that combine abundance, low cost, and environmental safety requirements that align perfectly with emerging energy harvesting approaches for flexible electronics applications [126]. Figure 6 outlines the strategy for developing and utilizing flexible LIG composites for energy harvesting systems (i.e., triboelectric generators and solar cells).

##### Triboelectric Nanogenerators (TENGs)

TENGs have revolutionized mechanical energy harvesting by leveraging contact electrification and electrostatic induction to efficiently convert ambient motion into usable electricity. These versatile devices combine high energy conversion efficiency with flexible design configurations, enabling diverse applications including micro-power systems, self-powered sensors, and control interfaces [127]. The operational principle involves relative motion between specially engineered triboelectric surfaces that create dynamic electric fields at electrode interfaces to ultimately generate an electrical output. Particularly promising is the integration of LIG into TENG architectures, where its exceptional conductivity, tunable porosity, and doping compatibility offer unprecedented opportunities for developing advanced flexible and wearable energy harvesting systems [128,129]. Material characterization of LIG-based TENGs employs comprehensive analytical techniques mentioned previously and includes ultraviolet photoelectron spectroscopy (UPS) used for deriving work function values. For performance evaluation, output measurements can utilize a controlled press/release system (function generator and power amplifier) to regulate frequency and force, while an oscilloscope records voltage output. Additionally, electrometer-based analyses provide capacitor charging profiles and cycle stability data through real-time monitoring of TENG electrical performance [21,130]. Several research groups have developed diverse fabrication strategies to create various types of TENG devices. Table 6 summarizes these approaches.

Stanford et al. investigated the performance of PI/LIG composite-based TENGs, demonstrating their effectiveness in both conductor-to-dielectric and fully dielectric (metal-free) configurations. The researchers achieved electrical outputs, with open-circuit voltages surpassing 3.5 kV and 8 mW peak power [131]. In another study, Chen and collaborators developed a laser manufacturing technique to fabricate monolithic multilayer carbon-based devices by exploiting the differential photoresponses of fluorinated ethylene propylene (FEP)/PI bilayer structures, enabling fluorine atom transfer from decomposed FEP. This approach allowed in situ growth of fluorine–LIG and LIG electrodes (top and bottom sides) to create a flexible Droplet Energy Generator, achieving 15.2 mW peak power (47.5 W m^−2^) from a single 105 µL water droplet falling from a 25 cm height. The device maintained 88% performance at 96% humidity, retained 70% power density after 10,000 cycles, and operated reliably across an extreme pH range (3–13) [34].

Recent research advances have also demonstrated that PI paper-based triboelectric nanogenerators (PIP-TENGs) significantly enhance production efficiency via laser-controlled fabrication. Moreover, by optimizing laser parameters, researchers developed foldable PIP-TENGs with tunable performance, achieving an impressive open-circuit voltage of 430 V (11.9 V cm^−2^) and peak power of 5 mW. Remarkably, an origami-structured TENG measuring 30 × 30 mm^2^ showed a 40-fold power enhancement, with voltage output increasing from 5.3 to 34.4 V cm^−2^. These advances have enabled practical applications, including smart footwear, intelligent leaves, matrix sensors, and responsive gloves, showcasing the technology’s potential for both energy harvesting and smart sensing solutions [132].

The success of the aforementioned applications hinges on innovative fabrication techniques. In this regard, De Oliveira and coworkers demonstrated an efficient approach for converting Kapton film into LIG and transferring it to PDMS, thus enabling simultaneous fabrication of interdigitated electrodes for microsupercapacitors and tribonegative layers with graphene-backed electrodes for TENGs. This integrated method facilitates the development of combined energy harvesting and energy storage systems suitable for powering small electronics. The LIG-based TENG fabricated by De Oliveira et al. achieved a 189.7 V open-circuit voltage, 39.8 μA short-circuit current, and 0.20 W m^−2^ power density [133].

Another group of researchers developed an innovative pressure sensor system by combining reduced graphene oxide (rGO) cloth with LIG through a simple process. The incorporation of LIG significantly enhanced the sensor’s performance, boosting pressure sensitivity from 20.6 kPa^−1^ to 30.3 kPa^−1^ while simultaneously improving the TENG’s charge transfer density from 160 µC m^−2^ to 270 µC m^−2^ [134]. Choi and colleagues achieved a significant breakthrough by developing a vertically aligned fibrous structure in their long carbon fiber-dominant LIG (LF-LIG) device. This architecture increased the effective contact area, thereby enhancing charge transfer efficiency. Remarkably, the LF-LIG structure demonstrated a 130-fold performance improvement (reaching 512 mW m^−2^) compared to conventional porous LIG-based TENGs [130]. Additionally, researchers have developed integrated energy systems using LIG electrodes fabricated through the one-step laser conversion of chitosan–lignin–boric acid films. The resulting 3D interconnected LIG networks produced single-electrode triboelectric nanogenerators (SE-TENGs) with PDMS dielectric layers, demonstrating reliable outputs (13.3 V, 1.7 μA) [21].

Additionally, researchers have demonstrated the potential of combining diverse fields, such as music and triboelectric energy harvesting through graphene-based TENG technology, suggesting new and promising opportunities for incorporating LIG. Alghami et al. developed advanced TENGs using Cellulose Nanocrystal (CNC) triboelectric layers paired with graphene electrodes to systematically investigate how CNC thickness and chemical functionalization affect performance metrics (i.e., output current, voltage, and power generation) when applied to piano playing. Their optimized design achieved an impressive power density of 0.4 W cm^−2^ when combined with polytetrafluoroethylene counter layers, along with exceptional durability, mechanical deformation stability, and a long-term performance consistency over three years [127].

Unfortunately, despite the significant advances for LIG-TENGs, critical challenges remain that hinder their widespread adoption. These devices demonstrate notable sensitivity to environmental humidity, with elevated moisture levels causing substantial charge dissipation and consequent power output reduction [135]. Additionally, operational durability is compromised by mechanical wear from repeated friction, leading to a progressive decline in charge density over extended use [136]. Perhaps most critically, the field lacks comprehensive studies examining temperature effects on performance, a fundamental gap that must be addressed to enable reliable commercialization and industrial implementation [135,136,137]. These persisting limitations in environmental stability, mechanical robustness, and thermal performance represent key barriers that must be overcome through targeted materials research and device engineering.

##### Photovoltaic (PV) Cells

Solar energy has risen in prominence as a leading sustainable solution to worldwide energy needs. Photovoltaic cells enable the direct conversion of sunlight into electricity through the photovoltaic effect, offering an environmentally friendly power solution. While solar technology presents significant advantages, its implementation faces two main limitations: intermittent availability of sunlight and relatively high initial equipment costs. These challenges continue to drive research into more efficient and affordable photovoltaic materials and systems to maximize solar energy utilization [126,138]. The three leading thin-film solar cell technologies, such as amorphous silicon (a-Si), copper indium gallium selenide (CIGS), and cadmium telluride (CdTe), each offer distinct advantages for photovoltaic applications [139]. On the other hand, graphene-based nanomaterials have garnered scientific attention for their tunable optoelectronic properties, making them highly promising for customized PV cells.

These advanced PV systems show potential for powering next-generation flexible and wearable electronics, including touch panels, LEDs, sensors, and high-speed FETs, offering a cost-effective alternative to conventional silicon-based solar cells. However, current graphene PV fabrication methods face significant challenges in processing efficiency and electrical performance, limiting their commercial viability. Key limitations include inconsistent material quality, scalability issues, and suboptimal charge transport characteristics that must be addressed to realize their full potential in renewable energy applications [140]. Photoluminescence and time-resolved photoluminescence measurements provide emission spectra data, while current–voltage measurements under simulated solar illumination quantify photovoltaic performance parameters such as efficiency, fill factor, and power conversion [140,141,142]. Table 7 provides a comparative overview of different LIG-based technologies for solar cell applications.

Speranza and collaborators fabricated a flexible LIG electrode; this device exhibited a catalytic activity for I_3_^−^/I^−^ reduction compared to costly Pt electrodes and outperformed Poly(3,4-ethylenedioxythiophene) (PEDOT) in reducing a copper bipyridyl complex electrolyte. When integrated into dye-sensitized solar cells (DSSCs), an open-circuit voltage of 0.94 V and a 60% efficiency boost over PEDOT-based cells was achieved (i.e., 3.08% to 4.96%). Additionally, the one-step laser process enabled the integration of LIG-DSSCs with SC, creating a flexible self-charging system that worked under both solar and indoor light (1500 lux), with storage efficiency increasing from 23% (1 sun) to 30% (low light) [143].

Renuka et al. followed another strategy, where they designed a flexible heterojunction PV device that was fabricated using ferroelectric Cr-doped BiFeO_3_ (BFCrO) as the energy-harvesting layer, sandwiched between p-NiO and n-WS_2_ window layers on a LIG electrode. Compared to a similar multi-junction PV built on indium–tin oxide (ITO), the LIG-based device demonstrated superior stability, flexibility, and conductivity, achieving a maximum PCE of 5.20%. Even after bending to 130°, the LIG electrode’s sheet resistance fully recovered, while ITO suffered from increased resistance due to cracking, highlighting LIG’s robustness for flexible optoelectronic applications [140]. Another study presented a one-step laser induction method to synthesize nitrogen and boron co-doped porous graphene (BN-LIG) as an efficient counter electrode for DSSCs. Electrochemical tests revealed enhanced conductivity and catalytic activity for triiodide reduction compared to single-doped variants, leading to a DSSC PCE of 4.99% [141]. Chu et al. demonstrated a one-step in situ synthesis of Pt-LIG composite through direct laser irradiation of a polyimide sheet coated with a low concentration of Pt precursor, creating an efficient Pt-minimized counter electrode for DSSCs. The resulting Pt-LIG-based DSSC achieved a PCE of 3.80%, matching the performance of conventional pure Pt-based DSSCs while significantly reducing platinum usage [144].

In another type of cell, Abdellatif et al. successfully fabricated a complete perovskite solar cell using LIG as a counter electrode, achieving a notable efficiency of 12.52%, a 1.4% improvement over conventional fluorine-doped tin oxide-based symmetric structures, despite a relatively high deviation in PCE (±7.6) [145]. Wang developed a one-step laser synthesis of LIG/NiOₓ composite electrodes to enable high-performance hole transport layer-free carbon perovskite solar cells. This approach creates compact electrodes without post-processing, yielding devices with a maximum of 14.46% PCE, 39% higher than conventional carbon electrodes (10.36%), through improved carbon–perovskite interfacial contact. The devices maintain 94% initial efficiency after 6 months in ambient air, far surpassing Spiro-OMeTAD/Au counterparts (78% retention after 12 weeks) [142].

LIG-based solar cells currently face significant challenges that must be addressed before commercialization. The primary limitations include relatively low power conversion efficiencies (PCEs < 15%) compared to commercial crystalline silicon modules (PCE > 21.5%) [139], as well as insufficient long-term stability under operational conditions. Critical knowledge gaps remain regarding device shelf life and performance degradation under environmental stressors such as temperature variations, humidity, and UV exposure. Successful integration of LIG technology into practical solar cell applications will require systematic solutions to these fundamental limitations, followed by demonstrations of scalable manufacturing processes to bridge the gap between laboratory prototypes and commercially viable products.

## 3. Mitigating Bacteriological Threats with LIG—Antibacterial Applications of LIG

### 3.1. Antimicrobial Remediation of Drinking and Wastewater

**Figure 7:**
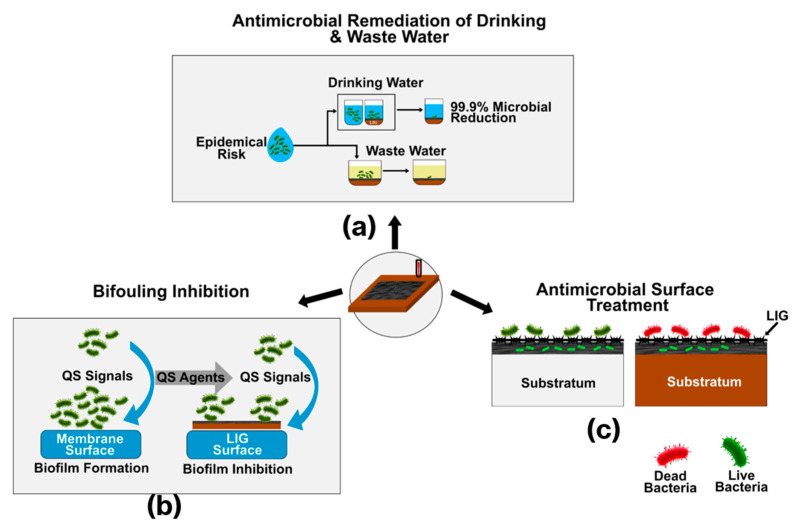
Antimicrobial Applications of LIG. (**a**, Top) Drinking and wastewater treatment: up to 99.9% microbial reduction can be achieved in water treatment and drinking water with LIG-based filtration. (**b**, Left) Biofouling inhibition. (**c**, Right) Surface coatings: LIG-functionalized substrates inhibit microbial survival on treated surfaces by microbicidal killing upon contact.

Numerous reviews have confirmed that waterborne pathogens, including viruses and bacteria, pose a recurring threat to public health, particularly in areas where water sources are untreated or inadequately treated [146,147,148]. LIG is being progressively studied as a novel material for water disinfection technologies, particularly as point-of-use filtration systems for drinking and wastewater treatment schemes, as it has described in Figure 7 [149]. According to studies, LIG filters made on polymer membranes or built into foam matrices are very effective at trapping and inactivating germs, resulting in reductions in bacterial load as high as 6-log when 5 V is passed through the filtration system [26]. LIG offers a great option for clean and environmentally friendly water filtration because it has intrinsic antibacterial properties and can be self-sterilized by Joule heating or sunlight irradiation [150]. Occasional heating of LIG due to its conductivity denatures bacterial cells and prevents biofilm formation [151]. Singh et al. showed that LIG electrodes have increased bactericidal activity upon electrical stimulation, taking advantage of electrochemical ROS production and bacterial membrane depolarization [151], while their porosity permitted effective water flow [23]. Electrochemical oxidation reactions on LIG surfaces have demonstrated the ability to kill microbiological contaminants along with sterilization via physical and electrical mechanisms [151,152,153]. Interestingly, LIG’s antiviral activity has also been validated in water treatment processes [154]. For example, Gu et al. prepared LIG with a positive surface charge (LIG^+^), which inactivated *Escherichia coli*, *Streptomyces*, and *Candida albicans* up to 100% within 10 min under sunlight irradiation, as well as an excellent antiviral activity toward human coronaviruses [155]. Barbhuiya et al. also illustrated that LIG filters could inactivate the Vaccinia virus when subjected to applied voltage with significant viral death at 20 V, though higher potentials were required for the inactivation of bacteria, suggesting a redox threshold difference across disinfection mechanisms [24]. These findings put LIG as not just an efficient antibacterial agent but as a multifaceted material capable of breaking up an extensive range of waterborne pathogens.

In addition to pathogen inactivation, researchers have tried to optimize the mechanical and functional characteristics of LIG so that it can be incorporated into water treatment membranes [13]. One of which is LIG-PVA composites prepared by Thakur et al., which showed ~99.9% bacterial rejection and superior antifouling performance due to the synergy between PVA’s film-forming stability and LIG’s conductivity [84]. Lasing parameters and choice of membrane support are also crucial in maintaining the structural integrity and function-specific application of LIG [156,157]. The significance of laser parameters and membrane support layers in maximizing filter performance was highlighted in the optimization of LIG synthesis on porous PES membranes by Misra et al., showing perfect 6-log bacteria inhibition at 5 V and efficient dye removal upon Joule heating [26]. Other innovations showing LIG material composites, surface modifications, and antimicrobial mechanisms are highlighted in Table 8.

Early studies are ongoing on the large-scale utilization of LIG [13]. However, there are some challenges to the effective utilization of LIG in large-scale or decentralized water schemes [164]. First, at off-grid locations, energy demands on Joule heating, particularly continuous operation, may be a constraint [165]. However, as highlighted by Barbhuiya et al. [166], Joule heating and the impact of stacking LIG composites on energy consumption may provide significant promises for energy conservation and address the rising water demand. By stacking a few heaters together vertically, LIG Joule heaters optimize water evaporation efficiency [166]. It was highlighted that this design increases the surface temperature, reduces thermal losses to the surroundings, and enhances heat transfer between LIG layers. Compared to using a single large-area heater, stacking LIG dramatically enhances evaporation rates and reduces energy requirements. For example, a triple stacking scheme utilized three times less power while generating seven times the amount of evaporation produced by an equivalent unstacked system. Second, LIG surface degradation with time can reduce performance and necessitate higher maintenance. Furthermore, it is unknown whether LIG-based filters work well in real-world complex water matrices (e.g., high turbidity and variable pH) since they are mostly studied in highly controlled laboratory settings [167]. Field trials in water-scarce and microbiologically contaminated areas should be performed in upcoming research to address the construction of gravity-driven or hybrid systems, combining LIG with renewable energy sources (like solar cells).

### 3.2. Biofouling Inhibition

Biofouling—the persistent buildup of biological material such as bacteria, proteins, and polysaccharides—is an essential threat to the efficiency and lifespan of marine structures, biomedical devices, and filtration systems [168,169]. Biofilm growth on membrane surfaces during water treatment and desalination reduces permeability, increases the cost of energy, and requires more frequent chemical cleanings, thereby compromising efficiency and sustainability [170,171]. Therefore, management of biofouling has evolved from being a maintenance matter to becoming a critical performance parameter in contemporary membrane technology and device design [172,173].

Researchers have created a few physical and chemical antifouling approaches, such as surface patterning, nanoparticle inclusion, and biocidal coatings, to reduce these effects [174,175,176]. Unfortunately, many of these processes are not long-lasting, are costly, and have unfavorable environmental implications [177,178]. LIG, however, has become a substitute antifouling material for use in this application and many others (e.g., membrane water filtration, biomedical sensing, and marine surface protection [179]) due to its active biofouling resistance brought on by its high electrical conductivity and tunable surface chemistry [8].

One of the most important and intricate steps in the creation of biofilms is bacterial adhesion to surfaces, which is greatly impacted by surface texture [180,181]. LIG utilizes its surface roughness, electrochemical activity, and the production of reactive oxygen species (ROS) to suppress biofilm growth [182]. While electrochemical stimulation substantially enhances bactericidal activity through the capacitive charge effect and possibly ROS formation [162]. Jashrapuria et al. illustrated that LIG surfaces also actively suppress microbial adhesion and biofilm growth in the absence of applied voltage due to their surface texture [183]. For marine environments, Manderfeld et al. demonstrated that LIG coatings on polymer substrates sustained plastrons, or surface-bound air layers, which prevented diatom (microscopic algae) adhesion. They also observed that the plastron-based surface protection could be dramatically extended by thermally regenerating these plastrons with Joule heating [184]. Moreover, hybrid membrane platforms that integrate LIG with polymer supports or GO provide multifunctional antifouling surfaces [185]. By combining textural and electrical effects, Thakur et al. synthesized LIG-GO ultrafiltration membranes that suppressed biofilm formation by 83% and achieved bacterial rejection efficiencies of 99.9% compared to conventional polymer membranes [23].

In addition to physical and electrochemical killing, LIG-based electroconductive membranes (ECMs) can also be used as real-time fouling sensors [186]. More significantly, composite LIG materials and functional coatings have introduced additional enhancements in antifouling applications [81]. For example, even under extended contact with *E. coli* and HeLa cells, Zambrano et al. attained greater than 90% sensor performance through zwitterionization of LIG electrodes against nonspecific protein and bacterial adhesion [187]. Similarly, Pisharody et al. integrated conductive LIG-coated feed spacers in spiral wound reverse osmosis modules, without compromising module life while preserving membrane integrity through prevention of biofilm formation and inactivation of *P. aeruginosa* under a fixed applied voltage (i.e., 12 V) [188].

Further development of an electrified LIG spacer made with inexpensive LIG carbon glue composite, like the one posited by Pisharody et al., can lead to the development of possibly novel electrically dependent anti-biofouling technologies in contrast to current biofouling management methods or procedures that call for repeated chemical cleaning and backwashing [188].

However, with the application of LIG in real-world, field conditions for fouling remain in its early stages [186]. A lot of research has been conducted for comparatively short periods and with static culture conditions [150]. It is not well understood how robust LIG will be when exposed to seawater, erosive fluxes, and aggressive cleaning cycles [167,189]. The effects on the environment of LIG degradation or separation from the environment must also be considered [149,150]. The investigation of stimuli-responsive LIG surfaces, which can self-clean with pH modulation or thermal pulse, can enhance their antifouling performance further [149]. To realize the full potential of LIG-based antifouling solutions, industrial designers, environmental scientists, and marine engineers will need to form interdisciplinary collaborations [190].

### 3.3. Antimicrobial Surface Treatments

Several researchers have demonstrated that often touched surfaces (e.g., consumer items, personal protective equipment, medical devices, building interiors, etc.) can serve as vectors of transmission for bacteria, and ultimately become sources for cross-contamination and hospital-acquired infections [191,192]. The creation of surfaces that sterilize bacteria upon contact and prevent microbial adhesion has become increasingly important considering the recurring emergence of antimicrobial-resistant bacteria [193,194].

In this context, LIG presents itself as an exciting prospect for the development of self-sterilizing surfaces based on its inherent conductivity, joule heating, photothermal behavior, and surface roughness-mediated antibacterial activity [149,159].

For example, M. G. Stanford et al. demonstrate the self-sterilizing ability of LIG against *E. coli* without chemical agents via joule heating [159]. Other researchers also demonstrate the effectiveness of self-sterilizing LIG-coated surfaces (e.g., hospital bed rails, door handles, face masks, and even clothing) against a wide spectrum of microorganisms upon exposure to sunlight or low-voltage Joule heating.

In addition to Joule heating, LIG also exhibits a photothermal bactericidal killing effect, which is an important property when used as a surface coating [195]. According to Huang et al., LIGs’ rapid photothermal conversion resulted in >99.998% inactivation of *E. coli* upon sunlight irradiation, suggesting a photothermal killing mechanism. They also demonstrated that >81% inhibition of bacteria occurred in the absence of heat. The authors credited this to LIGs’ intrinsic antibacterial activity, mediated by direct contact-dependent killing mechanisms [160].

Apart from thermal treatments, the surface properties of LIG play a major role in microbial death [179]. Luong et al. created LIG composites (LIGCs) based on cementitious and polymeric matrices to improve surface toughness and expand material compatibility [81]. Such multifunctional surfaces improved abrasion resistance and were incorporated into substrates like concrete, polyethylene, and polystyrene without loss of the antibacterial and photothermal LIG activity [81,196]. Temperature responsiveness, programmable hydrophobicity, and hierarchical roughness were added to the surfaces that were developed, which allowed for varied applications in consumer goods and building materials.

Despite this potential, a few challenges prevent the use of LIG-based antimicrobial surfaces in the real world [197]. Currently, nobody knows how long LIG will last after being scrubbed repeatedly, washed repeatedly, and targeted by disinfectants like bleach [198]. Moreover, a technical challenge is economically mass-producing LIG coatings or composites with reproducible performance [198]. Future studies must focus on creating LIG formulations that are wear-resistant, perhaps through multilayer structures or encapsulation, and evaluating to what extent these perform under field or clinical conditions [199]. To inform regulatory clearance, user uptake, and system integration, coordination with defense agencies and healthcare organizations will be necessary [149]. Furthermore, investigating the integration of LIG with heat signals or online microbial sensors would provide double-functional surfaces that can detect and destroy pathogenic threats at the same time it is required [200].

## 4. Mechanisms of Bacterial Death: Physical, Chemical, Electrical

**Figure 8:**
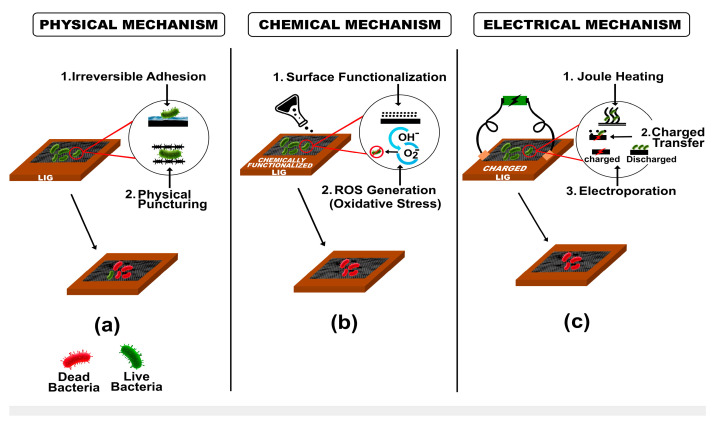
Mechanisms of Bacterial Death. (**a**) Physical—irreversible adhesion and puncturing; (**b**) chemical—surface modification and ROS generation leading to oxidative stress; and (**c**) electrical—Joule heating, charge transfer, and electroporation leading to cell integrity disruption.

LIG has been discovered to possess great potential in antibacterial applications owing to its exceptional chemical, electrical, and physical properties [4]. The production of ROS, chemical surface functionalization, electrical death, and physical destruction of bacterial cell membranes are some of the major mechanisms accountable for its efficiency in suppressing bacterial growth and promoting bacterial death (Figure 8) [201]. LIG presents a substitute for conventional antimicrobial drugs by mitigating the threats of a variety of bacterial infections through the varied mechanisms mentioned above, and thus minimizes the risk of acquiring antibiotic-resistant bacteria (an emerging contaminant) with time [150,152]. 

### 4.1. Physical Bactericidal Mechanisms

LIG, in many research works, has shown efficient physical antibacterial activity, mainly by contact-dependent action, based on its sharp edge morphology, surface roughness, hydrophilic nature, and propensity for high bacterial adhesion [202,203]. These characteristics enable LIG to effectively kill microorganisms.

The sharp edges within the LIG structure enable it to kill bacteria physically through mechanical stress and cell membrane puncture upon contact [8,204]. Once punctured, the intracellular contents of a bacterium leak out, ultimately killing the bacterium [204]. It has been demonstrated through studies that Gram-negative and Gram-positive bacteria are mechanically broken down by graphene nanospikes, with SEM images providing clear evidence of membrane rupture and cell lysis [205].

In addition to the physical mechanisms of microbial inactivation on LIG mentioned above, there has been the occurrence of irreversible bacterial adhesion to LIG surfaces. In this physical destruction mechanism, bacteria irreversibly and strongly adhere to the surface of LIG because of the extensive surface area of the material and the hydrophilic property of graphene [206]. This extended contact time may result in cell death, metabolic defects, and local membrane stress. Other bactericidal mechanisms, such as membrane disruption by the sharp edges of LIG, as well as oxidative or electrical actions, are enhanced through immobilization [207]. Due to the hydrophilic nature and rough texture of LIG, LIG has antimicrobial properties that not only prevent bacteria from attaching to the surface but also have a killing effect. Owing to the negatively charged LIG surface, foulants and also equally charged microorganisms do not stick to it [149]. These observations indicate that LIG’s antibacterial interactions are enabled by surface wetting behavior to a great extent.

Bacteria’s surface charge also plays a significant role in substrate/bacteria contact schemes, as strong electrostatic and van der Waals forces, according to Han et al., also augment mechanical stresses at the point of contact to amplify membrane disruption. Typically, being negatively charged because of compounds like teichoic acids or lipopolysaccharides, bacteria are attracted to positively charged materials [153,208]. This electrostatic attraction can increase the antibacterial effect of physical destruction mechanisms by increasing the frequency of bacterial contact with graphene surfaces [209].

Collectively, increasing the fineness and area of the sheets of graphene during LIG synthesis could be useful to improve the efficiency of physical death mechanisms (i.e., puncturing) [210]. It should be noted here, however, that the efficacy of physical disruption can vary based on the nature of the bacterial cell wall, given that the membrane composition of Gram-negative and Gram-positive bacteria differs, causing them to exhibit differences in sensitivity towards physical destruction [151]. Combining antimicrobial agents with LIG (i.e., surface functionalization) may also amplify its antimicrobial activity, especially against bacteria having more resistant cell walls [155].

### 4.2. Chemical Bactericidal Mechanisms

#### 4.2.1. Oxidative Stress

Chemically mediated oxidative stress is the process through which LIG displays potent antibacterial activity by producing ROS like hydrogen peroxide (H_2_O_2_), superoxide anions (O_2_^−^), and hydroxyl radicals (•OH) that target essential macromolecules in microbial cells, such as proteins, lipids, and nucleic acids [211]. Such targeting induces oxidative stress within the bacteria. As a part of such, microbial killing is caused by the irreversible cellular damage inflicted by ROS produced on or near the LIG surface.

The ability of LIG to generate ROS can be modulated by structural tuning. For example, tuning the porosity and edge structure of LIG directly enhanced electron density and ROS emission in a study by Wang et al., which showed 95.2% antimicrobial effect against *S. aureus* and 92.8% against *E. coli*. The higher effectiveness was explained by the authors as being due to the synergistic effect of membrane stress and oxidative stress [152]. Additionally, incorporating functional groups, such as oxygen, onto the surface of LIG can also induce oxidative stress within bacteria. Such chemical functionalization makes LIG more capable of catalyzing or generating ROS. The exact mechanism this antimicrobial technique utilizes is discussed in the surface functionalization section below.

When combined, these enhancements make oxidative stress a key chemical mechanism for bacterial death via LIG-based antimicrobial systems. Through structural tuning and surface functionalization, LIG can produce ROS in a calculated, efficient, and convenient manner [198]. In addition to the physical mechanisms of bactericidal action, this mechanism offers a chemical pathway to antimicrobial action, which extends the uses of LIG to industrial, environmental, and medical disinfection. Even with these encouraging results, challenges remain to be overcome in the pursuit of sustaining long-term ROS activity, material stability, and safety, as ROS are non-selective oxidants, so they can exert their action on human cells and tissues as well as on useful bacteria when deployed in real-world environments [150].

#### 4.2.2. Surface Functionalization

Surface functionalization is one of the best methods of improving LIG’s antibacterial activity [212]. This is accomplished by introducing chemical groups capable of directly interfering with bacteria, like nitrogen, oxygen, or metal-binding atoms, onto the LIG surface [207,213,214]. With the added ability to not leave toxic byproducts or need external energy, these surface alterations enable LIG to interfere chemically with the presence of bacteria, in addition to physically injuring them [215].

Copper-functionalized graphene is a perfect example. An extremely effective antibacterial surface was formed by Cu^2+^ ions bonded to graphene oxide, according to Zhang et al. Through a synergistic effect, the composite material killed bacteria more effectively than free copper ions and GO, as it showed over 95% bactericidal activity against *P. aeruginosa* and *S. aureus* [216]. High DNA damage, an increase in ROS levels, and physical disruption of bacterial cells were observed. Yang et al. reported another example by doping sulfur and molybdenum oxides into the LIG lattice. The doped material exhibited high antibacterial action, killing 99.9% of *S. aureus* and *E. coli.* Reactive oxygen species formed on the functionalized surface within this study were major contributors to this activity, oxidizing cellular components like proteins and lipids, and causing irreparable damage [163].

Incorporation of transition metals into the LIG surface to induce microbial death is another example of surface modification. In 2024, Sharma et al. designed a trimetallic LIG-based composite containing iron, zinc, and cobalt inside a metal–organic framework (Tri-MOF) embedded into LIG. At low concentrations of 0.91–4.54 mg/mL, this hybrid structure manifested an over 95% bacterial eradication efficacy with a minimum inhibitory concentration (MIC) of just 0.6 mg/mL against Gram-negative bacteria [158]. The researchers interpreted this result to mean the slow release of toxic metal ions responsible for oxidative stress within the bacterial cells that caused membrane rupture.

In addition, surface functionalization has also been used in antiviral and biosensing applications. To detect SARS-CoV-2 with unparalleled sensitivity, Brustoloni et al. functionalized LIG with gold nanostructures and tuned linker chemistry. The presence of metal ions and the chemical functional layers granted the surface passive antibacterial functionality, implying dual diagnostic and disinfection ability, although the main purpose was sensing [213].

Unfortunately, surface modification of LIG has several limitations, such as the instability and variable homogeneity of the functional groups that can lead to non-uniform antimicrobial activity over time or within varying environmental conditions [217]. While such changes are effective against various bacterial types, more work is needed to understand how sensitive and selective they are in acting against harmful bacteria only. To improve overall efficacy, particularly against biofilm-producing and antibiotic-resistant bacteria, research must be conducted to evaluate the synergistic mixture of surface modification with other antimicrobial processes like light activation or electrical killing [218].

### 4.3. Electrical Bactericidal Mechanisms

When exposed to electrical stimulation, LIG exhibits intense antibacterial activity via mechanisms like electron transfer, electrochemical disruption, and Joule heating. LIG can conductively kill bacteria by causing thermal and electrically induced stress, and electroporation-like damage [80,153,157,162,184]. These phenomena capitalize on LIG’s high electrical conductivity and surface reactivity. Single-layer LIG exhibits electricidal surface antibacterial activity by accumulating charge when an electric current is imposed and discharging said charge over time when the current is removed [153]. There are also several studies that have established that LIG is active against various pathogens, viruses, and Gram-positive and Gram-negative bacterial pathogens [154,219,220]. Using its conductive, porous character, LIG enables in-surface disinfection, as opposed to conventional electrochemical systems needing intricate electrode structures or high voltage [187]. LIG surfaces produce localized electric fields and ROS at applied voltages as low as 0.25 to 1.0 V [221]. These conditions kill bacterial membranes, cause oxidative stress, and block replication. This method is perfectly suited for integration since it enables reagent-free, on-demand disinfection.

**Figure 9:**
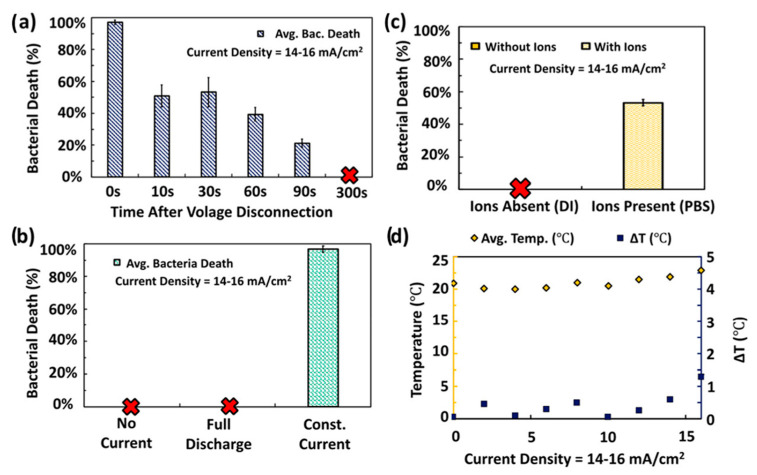
*Pseudomonas aeruginosa* electrically inactivated with a single LIG strip: (**a**) Bacterial death at various exposure times (0–300 s) following voltage disconnection, demonstrating a diminishing bactericidal effect as residual charge dissipates; (**b**) bacterial death under no current, full discharge, and constant current conditions; (**c**) the impact of ions on killing efficiency, showing that PBS killed more bacteria than DI water; and (**d**) the relationship between current density (14.6 mA/cm^2^) and the LIG strip’s surface temperature, indicating Joule heating contributions to antibacterial activity. Reprinted (adapted) with permission from [153]. Copyright 2023 American Chemical Society.

For example, Thakur et al. fabricated LIG-PVA membranes by synthesizing LIG and illustrating their feasibility in electrochemical disinfection devices in another work. Conductive LIG burst bacterial cells and had stable antibacterial properties during multiple cycles by facilitating electron transfer reactions at the membrane surface under low voltage [84]. The composite was especially well-fitted for continuous flow treatment of water processes due to its mechanical strength and electrical conductivity. Powell et al. also invoked bacterial death via electron transfer mechanisms via a “capacitive killing” strategy on a single film of LIG (log reduction of >4 for *P. aeruginosa*) without incurring Joule heating or other thermal effects, as shown in Figure 9. [153].

Broadly speaking, an argument can be made for implementing electricidal mechanisms into future generations of energy-efficient sterilizing technologies because of their low voltage requirement, robustness, and potential to be incorporated into membranes, filters, or fabrics. To do so, however, greater research efforts are needed that focus on streamlining material geometry, preventing overheating, and ensuring safety when applied in biomedical devices [215].

### 4.4. Thermal Bactericidal Mechanisms

LIG and its composites also show antibacterial activity via thermal mechanisms (e.g., photothermal or Joule heating). LIG can quickly convert electrical or light energy into heat, creating localized heating with temperatures high enough to inactivate viruses or kill bacteria [157,160,195,222]. Local LIG surface temperatures, through these thermal pathways, are elevated to levels that disrupt microbial membranes, denature critical proteins, and suppress DNA replication, all of which can irreversibly culminate in cell death [223]. For example, Huang et al. developed a LIG-based face mask that could self-report (i.e., create an electric signal that shows the extent of bacterial or particle deposition on its surface and photothermally enhance bacterial killing). Upon exposure to solar irradiation, the LIG layer on the mask reached temperatures of approximately 62 °C after prolonged exposures beyond 30 s and resulted in >99.998% inactivation of both *E. coli* and *S. aureus* within 10 min. Importantly, the study highlighted that this photothermal effect was obtained even when sunlight exposure was interrupted, making it applicable to real-world conditions [160]. The proposed mechanism of bacterial death was rapid membrane disruption, protein denaturation, and dehydration of bacterial cells due to the steep temperature gradient at the LIG interface.

As another example of the antibacterial prowess of LIG upon exposure to light/voltage, Sharma and Arnusch developed a LIG composite adhesive tape that could be heated by both electro- and photo-thermal means. The composite reached temperatures as high as 126 °C in 180 s under an applied voltage of 10 V and showed good response to solar irradiation with >70 °C increase under 120 mW/cm^2^. Upon testing against *P. aeruginosa*, the LIG tape showed 83% cell death under electrothermal conditions and as high as 99.99% under combined electro-/photo-thermal treatment. Surprisingly, bacterial death was evident even at temperatures around 35 °C due to the synergistic effects of infrared (IR), near-infrared (NIR) radiation, and electrical field stimulation [222].

Together, these studies demonstrate that the high thermal conductivity and rapid energy conversion of LIG render it a successful agent for non-chemical bacterial eradication. Future developments could include optimizing treatment duration, power input/efficiency, and layering regimes to better leverage the tradeoffs between thermal efficacy and reusability [223].

## 5. Conclusions

Laser-induced graphene is an economical, scalable, and eco-friendly approach to the synthesis of graphene when compared with traditional techniques. In this review, LIG has been discussed critically from its inception to applications, from synthesis conditions, including configurations of the laser, precursor materials, and doping, as well as the structural, chemical, and electrochemical characterization of LIG technology and its applications.

### 5.1. State-of-the-Art Advances in LIG

The state of the art in LIG research has evolved dramatically in recent years. For environmental applications, LIG-based technologies have been used for the detection and removal of poisonous HM elements like uranium, lead, and cadmium from water. With augmented electron transfer kinetics, LIG sensors exhibit ultralow limits of detection, and their integration into CDI and filtration devices has facilitated high-capacity selective metal adsorption. Decentralized water purification via these platforms is promising, particularly in resource-poor settings. Meanwhile, LIG has also showcased tremendous potential for energy applications. LIG is a material that offers rapid charge–discharge behavior, mechanical flexibility, and long-term electrochemical stability to be applied in supercapacitors, batteries, and even solar thermal devices. Structural optimization, metal oxide hybridization, and doping with heteroatoms have rendered its versatility, pushed its energy density, and expanded its use in low-energy electronics and environmental devices.

The antibacterial activity of LIG against a broad spectrum of bacterial species has also been reviewed. LIG was shown to kill over 99.99% of microorganisms in various forms via synergistic mechanisms such as electrically activated bactericidal killing, ROS production, and membrane disruption. Fabrication into textiles, membranes, and coatings creates new possibilities for self-sterilizing medical devices and surfaces.

### 5.2. Limitations of LIG

Each of these application areas still has limitations despite the many recent advances in each area. In sensing, reproducibility and stability are still concerns, even though LIG has enhanced electrochemical activity toward sensing and diagnostics. Long-term calibration protocols are not developed for LIG sensors, and temperature, humidity, and surface fouling can all impact sensor performance. Additionally, batch-to-batch variations in LIG synthesis can result in random selectivity and sensitivity of the sensors. In environmental sensing, though LIG is very sensitive and selective towards contaminants, its regeneration efficiency and adsorption capacity for long-term reuse in environmental filtering and sensing are still limited. Apart from the fact that the complexity of the environmental matrix may affect signal output, most LIG formulations synthesized to date lack the adequate chemical and mechanical stability required for applications that are commonly used. In energy harvesting, while LIG’s surface area and electrical conductivity are advantageous, its structural stability against repeated charge–discharge cycles makes it hard for it to be used in real applications of batteries and supercapacitors. Moreover, its commercial application is also restricted by its electrode–electrolyte interaction instability and low energy density when compared with traditional materials. Lastly, in antimicrobial applications, LIG’s long-term antibacterial activity under situations of continuous flow or biofouling remains a challenge. Robust toxicological and environmental assessments are also needed for authorization of biomedical or drinking water applications. Overall, cost and bulk material manufacturing cost scalabilities are among the biggest limitations to putting LIG into practical applications. The cost of powerful laser systems, as well as the resultant energy and maintenance demands that they entail, remains one of the major challenges of large-scale applications of LIG. Further research is needed to successfully address these issues in order to mitigate the concerns surrounding the scale-up and production costs of LIG.

### 5.3. Future Research and Directions

In general, future studies should focus on standardization of synthesis techniques for improved reproducibility, scalability, and minimized variability, enhancing structural integrity for filtration and energy applications, determination of environmental safety and toxicity to address legislative requirements for water purification and biomedical applications, and the development of multi-purpose composites for improving antibacterial longevity, selectivity, and durability to overcome these challenges.

However, despite these limitations, LIG is an ideal candidate for next-generation devices due to its versatility and scalability, and it will continue to need sustained interdisciplinary effort to connect fundamental research with engineering applications and overcome its current limitations. All in all, LIG has the potential to revolutionize major areas like renewable energy, environmental remediation, and health monitoring with concerted effort.

## Figures and Tables

**Table 1:** Performance Summary of the LIG-Based Sensor for Heavy Metal Detection.

Material	Analyte	LOD (μg L^−1^)	Range of Detection (μg L^−1^)	Interferences	Concentration or Ratio	Maximum RSD of the Peak Current by Interference Species (%)	Ref.
PI, LIG	Pb^2+^	0.5 (S/N = 3)	1–100	Cl^−^, CO_3_^2−^, H_2_PO^4−^, SO_4_^2−^, K^+^, Na^+^ or Zn^2+^	10 mg L^−1^ (all species)	8.23	[49]
PI, LIG/CuNP	Hg^2+^	2.41	2–30	Pb^2+^, Cd^2+^, Zn^2+^, Ni^2+^, Fe^2+^, Mn^2+^, and As^3+^	3.3, 23.6, 18.9, 12.9, 241.8, 25.2, 3.89 mg L^−1^, respectively	<5	[50]
PI, LIG/AuNP	As^3+^	0.18	0.2–1.0	Cu^2+^, Mg^2+^, Cd^2+^, Pb^2+^, Zn^2+^, Cr^2+^, Fe^2+^, Mn^2+^ and Ca^2+^	3 mg L^−1^ (all species)	<7	[37]
PI, LIG	Cd^2+^	0.01	0.1–160	Na^+^, Mg^2+^, Zn^2+^, Ca2^+^, SO_4_^2−^, NO^3−^, Cl^−^ and Pb^2+^	20 times higher	<2	[46]
PI, LIG	Pb^2+^ and Cd^2+^	0.4 (both) (S/N = 3)	0.5–20	-	-	-	[14]
PI, LIG/AgNP	Cd^2+^, Pb^2+^ and Cu^2+^	0.1 (S/N = 3)	20–120	Zn^2+^, Hg^2+^	400 (both ions)	0 (both ions)	[9]

**Table 2:** Immobilization of species through LIG.

Material	Immobilized Species	Maximum Adsorption Capacity mg g^−1^	Cyclic Performance of the Electrodes (Adsorption–Desorption Cycles)	Ref.
PI, LIG	Cd^2+^, Co^2+^ and Ni^2+^	3479.80, 1381.50, and 1448.70, respectively.	4 (all species)	[16]
PI, LIG/Co_4_S_3_)	U (VI) (UO_2_^2+^)	2702.79	6	[43]
PI, LIG/HTO	U (VI) (UO_2_^2+^)	1780.89	-	[17]
PI, LIG	La, Nd, and Ce	2510.50, 2349.25, and 2150.75, respectively.	5 (all species)	[74]

**Table 3:** LIG-based membranes for liquid matrices filtration.

Membrane	Removal or Treatment of the Target	Applied Performance Enhancement Technologies	Ref.
PES, LIG/CaCl_2_	Desalination	RF	[18]
PES, LIG/ACF/Phenol–formaldehyde	Water–oil separation	-	[19]
PES, LIG	Methylene blue dye and salt	IE, JH and solar drive	[26]
PES, LIG/glycerol	BSA	-	[20]
PES, LIG Janus/PDMS	Distillation	JH	[85]
PES, LIG	Iohexol and Cr (VI)	Electrochemical-based treatment	[80]
PES, LIG/PVA/GA	Wastewater (sludge, bioreactor feedwater)	-	[13]
PES, LIG/GO/GA	BSA	Electrochemical-based treatment	[23]
PES, LIG/PVA	BSA	-	[84]
PES, LIG	Wastewater	-	[27]

**Table 4:** Applications of LIG-based materials in battery technologies.

Material	Capacity	Active Material Loading (mg cm^−2^)	Thickness (µm)	CE (%)	Cycles	Current Density	Battery Type	Ref.
PI, LIG	280 μAh cm^−2^	0.1	40–60	99	100	0.100 mA cm^−2^	LIBs	[92]
PI, LIG	~160 mAh g^−1^	~15	-	99	250	~0.150 A g^−1^	LMBs	[96]
PI, LIG/NMnOMn_3_O_4_	992 mAh g^−1^	~0.8	-	67.3	35	0.200 A g^−1^	LIBs	[97]
PI, LIG/Al	115.7 mAh g^−1^	-	37	98.5	500	0.115 A g^−1^	LIBs	[98]
PI, LIG/SnS_2_	597 mAh g^−1^	1.0	34	~100	200	0.200 A g^−1^	SIBs	[99]
PI, LIG/MnOx	20 mAh cm^−2^	~7	-	100	3000	40.0 mA cm^−2^	LMBs	[100]
PI LIG/Ge	860 mAh g^−1^	0.25	~34	~60	2000	2.00 A g^−1^	LIBs	[93]

**Table 5:** Applications of LIG-based materials in SCs technologies.

Material	Areal Capacitance (mF cm^−2^)	Capacitance Retention % (Cycles)	Current Density (mA cm^−2^)	Equivalent Series Resistance (Ω)	Electrolyte	Ref.
PI/Co-MOF (ZIF-67), LIG	1.36	>99 (200,000)	1.0	430	PVA/H_2_SO_4_	[38]
PI, LIG/MoS_2_/MnS/Graphene	50.2	95.6 (10,000)	1.0	6.00	PVA/Na_2_SO_4_	[103]
PI, LIG/N	19.8	87.6 (10,000)	2.0	-	PVA/H_2_SO_4_	[104]
PI/H_3_BO_3_, LIG	65.7	~100 (50,000)	0.05	30.0	PVA/H_2_SO_4_	[105]
PI, LIG/Co_3_O_4_	10.9	97.8 (10,000)	0.08	150	PVA/H_2_SO_4_	[106]
PI, LIG/Fe_3_O_4_	644	74.0 (900)	1.0	-	PVA/H_2_SO_4_	[107]
PI, LIG (KOH activated)	32.0	95.73 (6000)	0.20	-	PVA/H_3_PO_4_	[108]
Poly (Ph-ddm), LIG	22.2	92.0 (10,000)	0.20	-	BMIM-BF_4_	[28]
PET, LIG/MoS_2_	35.3	88.7 (5000)	0.50	55.0	PVA/NaOH	[29]
PI, LIG/Co_3_O_4_^−^N	17.9	~70 (5000)	0.10	18.0	PVA/KOH	[109]
Carbon cloth -Gelatin ink, LIG/MoO_2_	81.8	85.1 (10,000)	1.0	64.9	PVA/H_3_PO_4_	[30]
PI, LIG/N/P	69.7	84.0 (10,000)	0.05	5.10	PVA/H_2_SO_4_	[110]
PI, LIG (Joule heating activated)	12.6	95.94 (10,000)	1.0	-	PVA/H_3_PO_4_	[111]
PI, LIG (KOH activated)	128	82.3 (5000)	0.2	94.6	PVA/H_2_SO_4_	[112]
Aramid paper, LIG	23.8	97.0 (10,000)	0.2	~27	PVA/H_2_SO_4_	[31]
PBO paper, LIG	46.3	87.0 (6000)	1.0	31.0	PVA/H_2_SO_4_	[113]
Parylene-C, LIG	1.66	96.0 (10,000)	0.50	-	PVA/H_2_SO_4_	[114]
Kevlar textile, LIG/P	125.3	88.0 (10,000)	0.10	18.9	PVA/H_2_SO_4_	[32]
Poly(furfuryl alcohol) (PFA)/Na_2_SO_4_, LIG/Na_2_SO_4_	9.7	103 (12,000)	0. 50	∼26	PVA/H_3_PO_4_	[115]
PI, LIG/Ag	1.20	88.0 (1500)	0.02	171	PVA/KOH	[116]
PI/FeCl_3_, LIG/Fe_3_O_4_	12.0	130 (4700)	1.0	129	PVA/H_2_SO_4_	[117]
Lignin/Na_2_SO_4_/SC(NH_2_)_2_, LIG/N/S	29.9	80.0 (5000)	1.0	69.8	PVA/LiClO_4_	[33]
PI/MoS_2_, LIG/MoS_2_	14.1	~90 (1000)	0.5	209	PVA/H_2_SO_4_	[118]
PI, LIG/Nickel ferrite (NFO)	198	97.0 (10,000)	1.5	10.4	PVA/KOH	[119]
PI, LIG/Ni	24.0	92.43 (5000)	0.30	39.9	PVA/KOH	[102]

**Table 6:** LIG-Based TENGs for Energy Harvesting.

Material	Open Circuit Voltage VOC (kV)	Peak Power Density (W m^−2^)	Peak Power (mW)	Load Resistance (MΩ)	Excitation Frequency (Hz)	Contact Area (cm^2^)	Ref.
PI, LIG, Aluminum	~3.50	2.40	~8.5	70.0	-	36.0	[131]
FEP/PI, LIG	~0.20	47.5	15.2	0.36	-	3.2	[34]
PI, LIG	0.43	-	5.0	7.0	5.0	36.0	[132]
PI, LIG	0.19	0.202	-	40.0	7.0	15.0	[133]
PI, LIG	-	512	-	5.0	1.0	1.0	[130]

**Table 7:** LIG-based devices for solar cell technology.

Material	Power Conversion Efficiency (PCE) (%)	Ref.
PI, LIG	4.96	[143]
PI, LIG/WS_2_/BFCrO/NiO/Ag	5.20	[140]
Thermoplastic polyimide (TPI)/H_3_BO_3_, LIG/BN	4.99	[141]
TPI, LIG/N	4.59	[141]
PI, LIG	3.80	[144]
PI, LIG	12.5	[145]
Polybenzimidazole (PBI) and Ni(acac)_2_, LIG/NiO_X_	14.5	[142]

**Table 8:** Overview of Application of LIG and Antimicrobial Mechanisms.

Material	Antimicrobial Mechanisms	Surface Modification	Effective Voltage	Microbe	Antimicrobial Effect	Ref.
LIG/Tri-MOF	Capture killing.	Trimetallic Metal–Organic Framework (Tri-MOF)	-	*P. aeruginosa*	≥95%	[158]
LIGP	Combined membrane stress and (ROS).	Oxygen Plasma	-	*E. coli* *S. aureus*	92.8% 95.2%	[152]
LIGC (LIG Composite)	Electrical effects (Joule heating); surface biofilm resistance.	Electrically Enhanced + Textured	2.5 V	Mixed bacteria	~6-log reduction at 2.5 V	[81]
LIG, P-LIG-B, P-LIG-S, P-LIG-SO.	Physical and electrical contact of the bacterial cells with the surfaces.	Electrically Enhanced.	1.1–2.5V	*P. aeruginosa* *E. coli*	99.9%	[151]
LIG	Delayed LIG exposure time after applied voltage disconnection (capacitive killing).	Electrically Enhanced	-	*P. aeruginosa*	∼ 97%	[153]
LIG	Temperature-dependent killing.	Joule Heating	-	Airborne Bacteria	100%	[159]
LIG	Physical disruption and photothermal heating.	-	-	*E. coli* *S. epidermidis*	99.998%	[160]
LIG LIG + ZnO LIG + ZnOAg	Zn^2+^ release and electrostatic interactions, synergistic anti-bactericidal effect between Ag and ZnO.	ZnO and Ag-Doped ZnO Nanocrystals	-	*E. coli* *S. aureus*	∼100% 84%	[161]
LIG	Electroporation via stored charge discharge. Capacitance-driven charge transfer.	-	1 to 2 V	*E. coli* *S. aureus*	100% (at 2V)	[162]
MoOx/Sulfur-Doped Laser-Induced Graphene (MSLIG)	ROS generation. Photothermal effect. Electrostatic repulsion. Physical damage from sharp edges.	Sulfur Doping and MoOx Nanoparticle Deposition.	-	*E. coli* *S. aureus*	~87% reduction in viability after 4 h.	[163]
